# Empowering Global AMR Research Community: Interactive GIS dashboards for AMR data analysis and informed decision-making

**DOI:** 10.12688/wellcomeopenres.21010.2

**Published:** 2024-09-03

**Authors:** Stephen Obol Opiyo, Racheal Nalunkuma, Stella Maris Nanyonga, Nathan Mugenyi, Andrew Marvin Kanyike

**Affiliations:** 1 Patira Data Science, LLC, Westerville, Ohio, 43081, USA; 2 Mengo Hospital, Kampala, Central Region, Uganda; 3 University of Oxford, Oxford, England, UK; 4 Faculty of Medicine, Mbarara University of Science and Technology, Mbarara, Uganda

**Keywords:** Antimicrobial Resistance (AMR), Geographic Information System (GIS) dashboards, Data analysis, Informed decision making

## Abstract

**Background:**

Antimicrobial Resistance (AMR) is a critical global public health concern, demanding effective tools for research, data analysis, and decision-making. This study proposes a groundbreaking approach to empower the global AMR research community by introducing interactive Geographic Information System (GIS) dashboards. These dashboards aim to facilitate comprehensive data analysis of AMR across multiple countries, providing insights into antimicrobial usage (AMU), resistance patterns, and geographic distribution.

**Methods:**

The approach involves developing GIS dashboards that integrate and harmonize AMR data from diverse sources, including clinical laboratories, surveillance networks, and public health agencies. A comprehensive dashboard was created to encompass data from multiple countries, providing advanced analysis and visualization tools. Another dashboard was developed with a specific focus on Kenya and Uganda to enable comparative analysis of AMR within these regions. Additionally, a simulated dataset for Kampala, Uganda, was generated to address data gaps in this area.

**Results:**

The GIS dashboards serve as powerful tools for visualizing and analyzing AMR-related datasets, providing stakeholders with a comprehensive view of the global AMR landscape. These dashboards offer valuable insights into antimicrobial usage, resistance patterns, and geographical distribution. The centralized platform facilitates data exploration and analysis, aiding researchers, policymakers, and healthcare professionals in making informed decisions to combat AMR.

**Conclusions:**

In conclusion, the study demonstrates that the developed GIS dashboards empower stakeholders by providing valuable insights and informed decision-making capabilities. The dashboards serve as essential tools for addressing the global challenge of AMR, allowing for a deeper understanding of the problem and informing effective strategies. The approach outlined in this study has the potential to significantly contribute to the ongoing efforts to combat AMR on a global scale.

## Introduction

AMR stands as a formidable global challenge, deeply intertwined with the framework of public health and healthcare systems across the world ^1^ The One Health concept, which recognizes the interconnectedness of human, animal, and environmental health, is particularly vital in understanding AMR, as it bridges the gap between healthcare systems and agricultural practices where antibiotic use is common ^2^. This approach is essential in sub-Saharan Africa, where the convergence of human and animal health systems heightens the risk of resistant pathogens spreading across sectors. Such regions often grapple with limited resources, inadequate healthcare infrastructure, and widespread antibiotic use in both medical and agricultural settings. These factors make the One Health framework especially critical in addressing AMR's multi-sectoral impact ^1^. Despite AMR's significant burden in sub-Saharan Africa, data on the region's AMR rates remain scarce. There is an urgent need to improve antibiotic stewardship and create context-specific treatment guidelines, particularly in East Africa, where high rates of microbiologically confirmed urinary tract infections (UTIs) are driven largely by multidrug-resistant (MDR) Escherichia coli and Staphylococcus spp. ^3^.

Research spotlighting antibiotic usage in hospitalized neonates reveals elevated mortality rates linked to culture-positive sepsis and notable antibiotic resistance ^4^. Diverse antibiotic regimens, often divergent from global guidelines, underscore the need for pragmatic trials to identify effective antibiotic protocols in regions grappling with substantial AMR challenges. Attention shifts to peri-urban smallholder poultry systems in Kenya, shedding light on prevalent antibiotic use in farming practices without prescriptions, prompting concerns about AMR and drug quality, necessitating interventions to promote responsible antibiotic use ^5^. Studies examining hurdles in implementing antimicrobial use (AMU) surveillance in Tanzania and Uganda highlight issues such as poor data quality, limited digitalization, and inadequate resources, underscoring the necessity for capacity building and continuous quality improvement ^6^. The evolution of national AMR surveillance systems in African countries from 2017 to 2019 is discussed, emphasizing constraints like limited laboratory capacity and staffing ^7^. A sustained increase in resistance among commonly used antibiotics in surgical wards of a Ugandan hospital from 2014 to 2018 underscores the need for ongoing monitoring, surveillance, and infection prevention and control practices ^8^.

The introduction of the One Health Evaluation of Antimicrobial Use and Resistance Surveillance (OHE-AMURS) tool allows for a detailed assessment of progress in integrated AMR and AMU surveillance in Canada, facilitating a systematic evaluation of the complex surveillance system ^9^. Progress and challenges in achieving comprehensive, integrated AMR/AMU surveillance in Canada are discussed, highlighting persistent gaps and the urgent need for policy changes, increased resources, and prioritization by government entities ^10^. An examination of AMR surveillance in Nepal reveals challenges in data reporting and the adoption of digital tools to enhance accuracy, with standardized data contributing to a One Health AMR surveillance portal, enabling evidence-based decision-making ^11^. Addressing the multifaceted AMR requires an integrated approach. We expressed interest of working with AMR data and submitted an abstract ^12^ for Pfizer Atlas dataset ^13^.

## Objectives

### 2023_06_15 atlas_antibiotics.csv dataset

Upon receiving access to the dataset, we promptly downloaded it and performed a preliminary descriptive statistical analysis to obtain an overview of its contents. However, we encountered several challenges during our analysis. The dataset had limited data from African countries, making it difficult to generalize findings across the continent. Furthermore, the available data from African countries covered less than five years, reducing its usefulness for comprehensive analysis. Although we discovered a dataset from Uganda, it only contained information for 2021, while the dataset for Kenya spanned 2013, 2014, and 2021. Consequently, we adjusted our research approach to develop tools that address these challenges and identify patterns of AMR, benefiting future researchers’, health professionals, and policymakers beyond this competition. To fulfill this aim, we outlined the following objectives:

***Objective 1.*** Develop an interactive Geographic Information System (GIS) dashboard encompassing all countries within the Vivli dataset. This dashboard offers a comprehensive data analysis platform for researchers, policymakers, and health professionals to gain valuable insights across various countries.

***Objective 2.*** Create an interactive GIS dashboard specific to the Vivli datasets from Kenya and Uganda. This focused dashboard would provide researchers, policymakers, and health professionals with a more detailed view of the datasets from these two countries, allowing for comparative analysis and targeted investigations.

***Objective 3.*** Generate a simulated dataset, such as for Kampala, Uganda. This dataset will help augment the limited data available, thereby expanding the scope of analysis for researchers, policymakers, and health professionals interested in studying AMR patterns in the region.

By accomplishing these objectives, we aimed to develop dashboards that would serve as valuable tools for researchers, policymakers, and healthcare professionals. Ultimately, our goal is to provide a comprehensive solution that could be utilized beyond the scope of this competition. Our dashboards provide realtime updates, customized visualization of AMR data and user friendly interfaces.

## Methods

### Descriptive statistical analysis

The dataset underwent descriptive statistics analysis using the R ^14^ statistical software version 4.2.2.

### Chi-square test of proportions

A chi-square test of proportions was performed to examine the distribution of isolates across different regions, including Africa, Asia, Europe, Latin America and the Caribbean, Middle East, and North America. Additionally, another chi-square test was performed to assess the distribution of years of available number dataset across these regions.

### Identification of coordinates

To develop an interactive GIS dashboard, it is essential to have latitude and longitude positions for each country. To accomplish this, a coordinate was assigned to the capital city of each country, utilizing information from Wikipedia ^15^. These coordinates were then incorporated into the dataset, enabling accurate geographical representation and visualization within the dashboards.

### Development of GIS web-based dashboards

The dashboards were developed using online ArcGIS from Esri ^16^. The dashboards at Esri are GIS-based and utilize web technology. They are constructed in the cloud, leveraging Esri's platform, and rely on datasets containing latitude and longitude coordinates.

## Results

### Descriptive statistical analysis

The dataset analyzed in this study consists of 83 countries and a total of 863,509 isolates. It encompasses 126 variables and includes information on 345 species. Table 1 provides an overview of the distribution of isolates across different regions and the availability of data over the years. The African region has the lowest number of datasets and the fewest years of available data, while Europe has the highest numbers. Chi-square tests confirmed that both the proportion of isolates in the region and the proportion of countries with at least 9 years of data significantly deviate from the expected proportion of 0.17 (p < 0.001, Table 2 and Table 3).

**Table 1.** Descriptive statistic results of the Atlas dataset as of June 15 ^th^, 2023: The dataset contains a total of 863,509 isolates. To determine the proportion, the number of isolates in each region is divided by the total number of isolates. Europe has the most extensive collection of datasets and the longest historical data, contrasting sharply with Africa, which possesses the least abundant dataset count and the shortest temporal coverage.

	Africa	Asia	Latin America and Caribbean	Middle East	Europe	North America
Number of isolates	22,717	116,845	96,309	37,112	419,496	171,030
Proportion of isolates per region	0.03	0.14	0.11	0.04	0.49	0.20

**Table 2.** The Atlas dataset, as of June 15th, 2023, shows the distribution of countries with 18 years of available data from 2004 to 2021. We set a criterion where each country should have at least 9 years of data out of the total 18-year period. The proportion was calculated by dividing the number of countries with at least 9 years of data by the total number of countries in each region. Isolates are unevenly distributed across various regions, such as Africa, Asia, Europe, Latin America, and the Caribbean, with Africa having only 13 representatives.

	Africa	Asia	Latin America and Caribbean	Middle East	Europe	North America
Number of countries	13	14	15	8	31	2
Number of countries with at least 9 years of dataset	2	11	8	4	23	2
Proportion of countries with at least 9 years of dataset	0.15	0.79	0.53	0.50	0.74	1.00

**Table 3.** Chi-square tests of proportions for the distribution of isolates across regions, and of distribution of countries with at least 9 years of dataset. The availability of dataset years varies among different regions, encompassing Africa, Asia, Europe, Latin America, and the Caribbean, with Africa exhibiting the shortest duration of available data.

	Chi-square	Df	p-value
Proportion of isolates per region	85.713	5	< 0.001
Proportion of countries with at least 9 years of dataset	69.717	5	< 0.001

### Development of web-based interactive GIS dashboards

**Figure 1.**
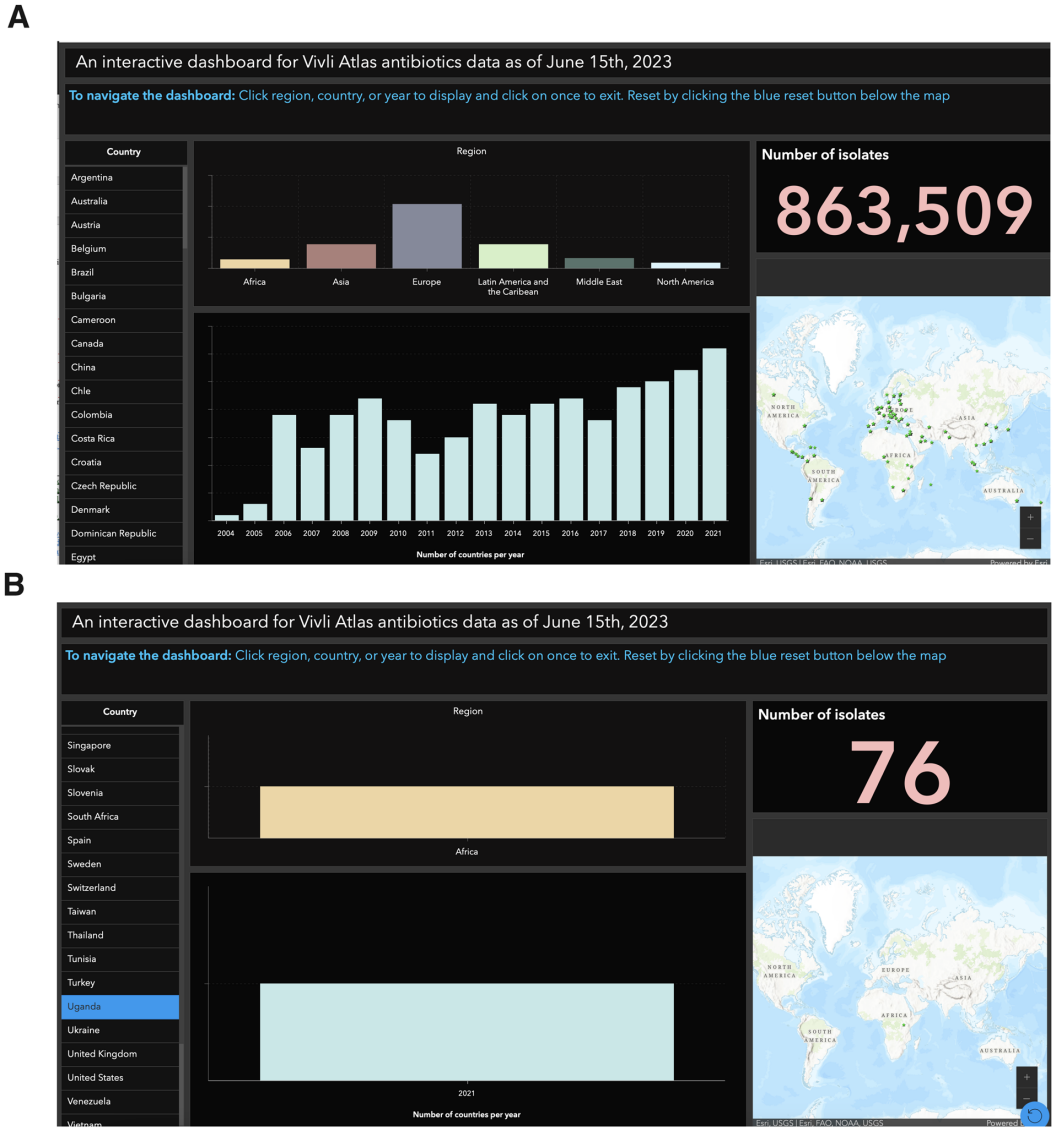
Interactive GIS dashboards: **A**) for all countries, **B**) for Uganda. Figure 1 **A** presents a comprehensive dashboard tailored for researchers, policymakers, and healthcare professionals, providing tools like a map, country selection, regional breakdown, and clear navigation instructions for analyzing and exploring data across multiple countries. Meanwhile, Figure 1 **B** offers a visually appealing presentation with a focus on Uganda, simplifying data interpretation, aiding monitoring and evaluation efforts, and providing comprehensive information for hypothesis formulation, particularly emphasizing the examination of the number of isolates and data collection duration in the country.

***Objective 1. Develop an interactive Geographic Information System (GIS) dashboard encompassing all countries.*** This dashboard is a valuable tool for researchers, policymakers, and healthcare professionals to explore and analyze data across multiple countries. It offers insights on various aspects, including a map, country selection, regional breakdown, number of isolates, number of countries per year, and navigation instructions ( Figure 1A). To examine the number of isolates in Uganda and the data collection duration, simply go to the "Country" section, find Uganda, and click on it. This will display only Uganda's data, removing other countries from view. The dashboard's visually appealing presentation simplifies data interpretation, aids monitoring and evaluation efforts, and provides comprehensive information for hypothesis formulation ( Figure 1B). The dashboard can be accessed using the link ^17^.

**Figure 2.**
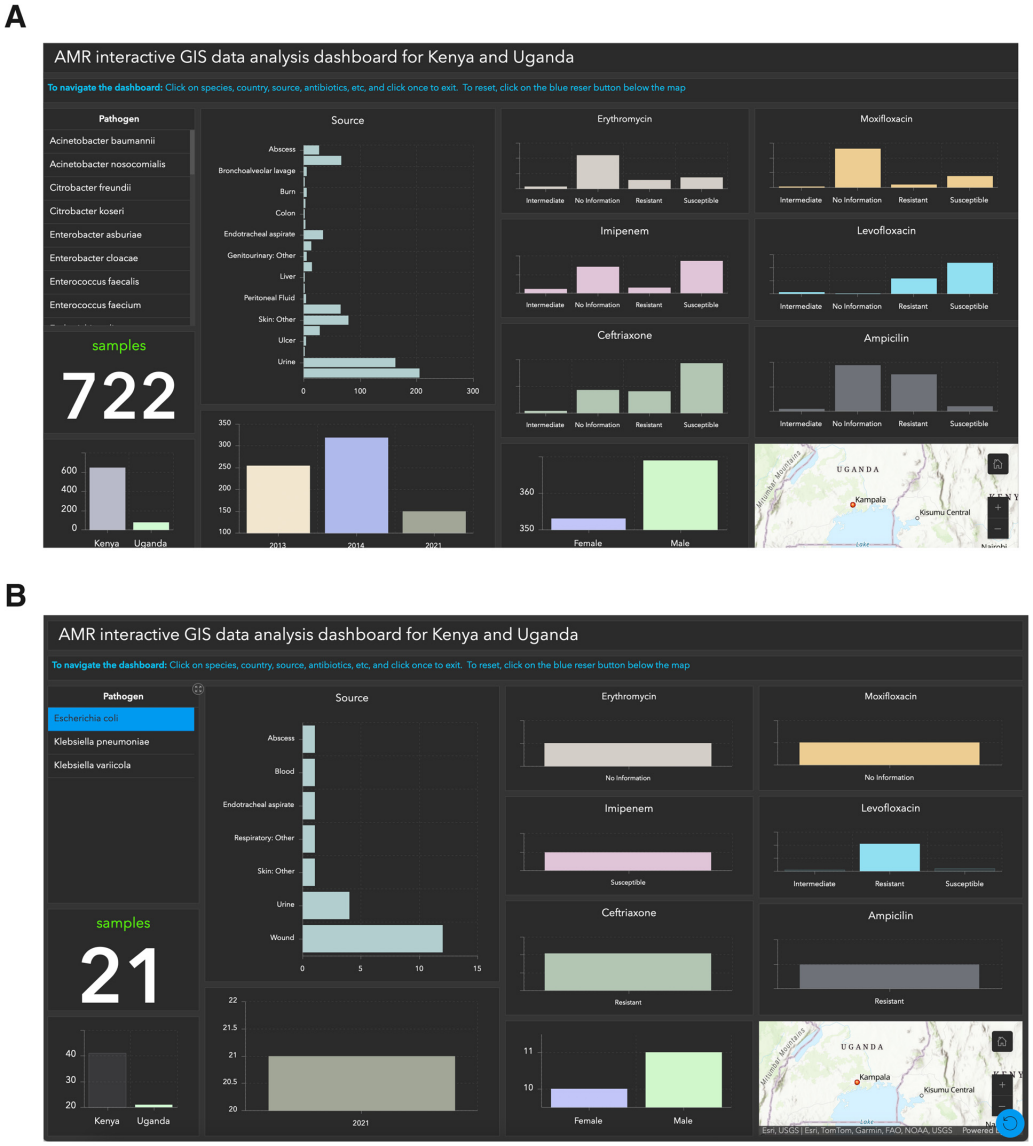
Interactive GIS dashboards: **A**) Kenya and Uganda, **B**) Performance of antibiotics on *E.coli.* Figure 2 **A** provides a comprehensive dashboard overview, highlighting key data points such as species, country, sample sources, count, gender, and antibiotics. Figure 2 **B** illustrates antibiotic resistance in E. coli samples from Uganda, revealing resistance to Ampicillin, Levofloxacin, and Ciprofloxacin in 21 samples, while all samples displayed susceptibility to Imipenem.

***Objective 2. Create an interactive GIS dashboard specific to the datasets from Kenya and Uganda*.** The specialized dashboard aims to provide a comprehensive and detailed perspective on data from two specific nations. Its main purpose is to empower researchers, policymakers, and healthcare professionals by facilitating comparative analysis and focused investigations. The dashboard offers a comprehensive overview of data points such as species, country, sample sources, sample count, gender, and antibiotics ( Figure 2A). Users can apply filters based on these variables. For example, Figure 2B illustrates the antibiotic resistance of *E.coli* samples from Uganda. Ampicillin, Levofloxacin, and Ciprofloxacin were ineffective in 21 samples (Resistant), while all samples responded positively to Imipenem (Susceptible). The interactive dashboard simplifies the visualization of antibiotic response, making analysis more accessible. Access the dashboard via the provided link ^18^.

**Figure 3.**
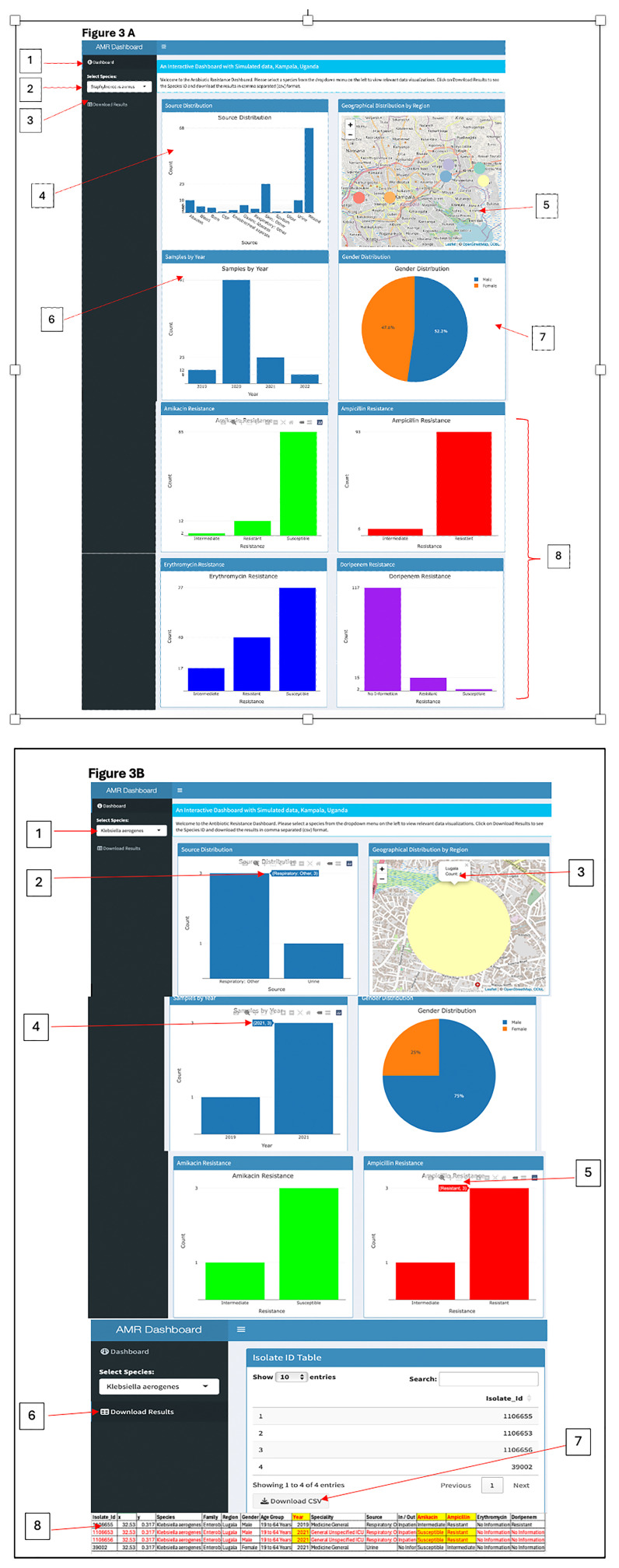
Interactive GIS dashboards: **A**) simulated Kampala data, **B**) Klebsiella aerogenes from only Lugala. Figure 3 **A** displays the simulated data examining various regions within Kampala, while Figure 3 **B** highlights the 2019 and 2021 detection of Klebsiella aerogenes exclusively in Lugala, revealing three resistance to Ampicillin but susceptibility to Amikacin. This simulated dataset, offers crucial insights into AMR trends and can be used to empower stakeholders for more informed decision-making.

***Objective 3. Generate a simulated dataset specifically for Kampala, Uganda.*** To apply information gained from the two previous dashboards discussed above we simulated a dataset, for researchers, policymakers, and healthcare professionals studying AMR patterns in Kampala, Uganda, it is crucial to create a comprehensive and representative dataset for the region. This dataset would fill the existing data gaps and provide valuable insights, empowering stakeholders with a broader understanding of AMR trends and enabling them to make more informed decisions. In our simulated data, we examined various regions within Kampala ( Figure 3). The dashboard [1], illustrated in Figure 3A, provides a comprehensive and interactive visualization of antibiotic resistance data using the R Shiny Dashboard framework ^19^. At the top, a welcome message instructs: “Welcome to the Antibiotic Resistance Dashboard. Please select a species from the dropdown menu on the left to view relevant data visualizations. Click on 'Download Results' to access the Species ID and download the data in CSV format.” At the top left [2], users can choose from various species via a dropdown menu, with "Staphylococcus aureus" selected by default. Below this selection is the "Download Results" button [3], allowing users to export data in CSV format. The Source Distribution plot [4] showcases the count of samples across different sources such as "Abscess”, “Blood”,” Burn”, and others. To the right of the source plot, a Geographical Distribution map [5] displays regional markers across Kampala, with interactive hover functionality revealing the name of each region and clickable markers that provide the total sample count for that region. Additional visualizations include the Samples by Year bar plot [6], which depicts the number of samples collected during the years 2019, 2020, 2021, and 2022. A Gender Distribution pie chart [7] illustrates the percentage of male and female participants, with a click feature revealing both the count and percentage values. Lastly, the Antibiotic Resistance plots [8] visualize resistance levels for antibiotics such as Amikacin (green) and Ampicillin (red), categorized into Intermediate, Resistant, Susceptible, or lacking information. Access the dashboard via the provided link ^20^

When " *Klebsiella aerogenes*" is selected from the species dropdown ( Figure 3B), the dashboard dynamically updates to display *K. aerogenes* visualizations. The Source Distribution Chart indicates that samples were collected from “Respiratory: Other” (3 samples) and “Urine” (1 sample). The Geographical Distribution Map pinpoints Lugala as the sole location of *K. aerogenes* cases, with a total of four cases reported. Additional insights are provided by the Samples by Year Plot, which shows an increase in sample counts over time: 1 sample in 2019, 1 sample in 2020, and 2 samples in 2021. The Antibiotic Resistance Charts reveal full resistance to Ampicillin (red, 4 cases) but full susceptibility to Amikacin (green, 4 cases). Users can export the filtered data using the "Download Results" button, which generates a CSV file. Notably, in 2021, K. aerogenes demonstrated resistance to Ampicillin (2 cases) while remaining susceptible to Amikacin (2 cases). Access the R Shiny dashboard via the provided link ^20^.

### Impact of the work

The GIS web-based dashboards described are comprehensive and versatile tools designed to assist researchers, policymakers, and healthcare professionals in their AMR data exploration and analysis efforts. They offer a wide range of functionalities and aims to provide valuable insights specifically focused on multiple countries. By utilizing the dashboards, users can access and examine various datasets relevant to their respective fields. These datasets may include information related to healthcare, public health, demographics, economics, and other relevant factors. The dashboard acts as a centralized hub, aggregating and organizing these datasets for easy access and analysis. Researchers can leverage the dashboards to delve into the available data, perform complex queries, and conduct in-depth analyses. They can explore patterns, trends, and correlations across different countries, facilitating cross-country comparisons and enabling the identification of similarities and differences. Policymakers can benefit from the dashboards by utilizing the insights gained from the data to inform their decision-making processes. They can examine the impact of different policies or interventions in specific countries, evaluate the effectiveness of existing strategies, and identify areas that require attention or improvement. Healthcare professionals can also leverage the dashboards to gain a better understanding of healthcare systems, AMR prevalence, healthcare outcomes, and other relevant metrics across multiple countries. This knowledge can contribute to the development of evidence-based practices, policies, and interventions aimed at improving healthcare delivery and patient outcomes. Overall, these dashboards act as powerful tools that empower researchers, policymakers, and healthcare professionals to explore, analyze, and derive valuable insights from available AMR datasets pertaining to multiple countries. Its broad scope and functionality make them valuable assets in the pursuit of knowledge, evidence-based decision-making, and improvement of AMR research worldwide.

## Conclusion and limitations

The development of interactive GIS Dashboards represents a significant advancement in empowering the global AMR Research community. By providing comprehensive data analysis capabilities and facilitating informed decision making processes, these dashboards contribute to the fight against AMR on a global scale. However, the research confronts notable challenges, particularly the scarcity of comprehensive AMR data in Africa. Limited sample sizes, such as those in Uganda and Kenya, may not accurately reflect the full spectrum of resistance patterns. Furthermore, inconsistent data collection and reporting across Africa reduce the reliability and generalizability of dashboards, particularly for Uganda and Kenya. The study underscores the necessity for standardized data collection, enhanced laboratory capabilities, and increased investment in healthcare infrastructure to fully leverage dashboards in the fight against AMR in Africa

## Ethics and consent

Ethical approval and consent were not required.

## Data Availability

The data for objectives 1 and 2 cannot be made available as it is under license by a third party. However, the dataset can be requested from https://github.com/so13839/Team-8952-Vivli-AMR-Competation/blob/main/Vivli_Intial_abstract.pdf ^13^ using the following information:- Data contributor name: Pfizer (AMR); DOI: https://doi.org/10.25934/PR00007930; AMR ID: VIV00007930; and Dataset ID: ATLAS_Antibiotics. The simulated data for Kampala, Uganda used in objective 3 is available to the public. The team composed of participants from Uganda, United States, and United Kingdom obtained data for Vivli AMR Surveillance Open Data Re-use Data Challenge from Vivli ^13^. This publication is based on research using data from Pfizer, obtained through ^13^. The dashboard creation codes for Objectives 1 and 2 are not available, as the dashboards were originally constructed using the graphical user interface (GUI) on ArcGIS. To replicate the dashboards, we can leverage open-source QGIS GIS software ( https://qgis.org/en/site/), utilizing the information outlined in the materials and methods section. The R Shiny app call “app.R” for creating dashboard for Objective 3 is available at GitHub at ^21^, and information about the Shiny app and instructions for installing the app is available in RMarkdown format, pdf format, and html format at GitHub at 22 The dataset is for Objectives 1 and 2 conveniently formatted in CSV, and city coordinates can be obtained from Wikipedia. The process involves recreating the dashboards using QGIS, drawing from the provided materials and methods, and utilizing the CSV dataset along with city coordinates. The simulated dataset “Kampala_data.csv”) for the Objective 3 is available in a CSV format from GitHub at 23
