# Empowering Global AMR Research Community: Interactive GIS dashboards for AMR data analysis and informed decision-making

**DOI:** 10.12688/wellcomeopenres.21010.4

**Published:** 2025-03-07

**Authors:** Stephen Obol Opiyo, Racheal Nalunkuma, Stella Maris Nanyonga, Nathan Mugenyi, Andrew Marvin Kanyike

**Affiliations:** 1 Patira Data Science, LLC, Westerville, Ohio, 43081, USA; 2 Fellow of Uganda National Academy of Sciences, Kampala, Uganda; 3 Ohio State University, Columbus, Ohio, 43210, USA; 4 Mengo Hospital, Kampala, Central Region, Uganda; 5 University of Oxford, Oxford, England, UK; 6 Faculty of Medicine, Mbarara University of Science and Technology, Mbarara, Uganda

**Keywords:** Antimicrobial Resistance (AMR), Geographic Information System (GIS) dashboards, Data analysis, Informed decision making

## Abstract

**Background:**

Antimicrobial Resistance (AMR) poses a global public health challenge, necessitating advanced tools to support data analysis, and visualization. This study introduces interactive Geographic Information System (GIS) dashboards as innovative platforms for AMR data analysis and visualization, offering comprehensive insights into resistance patterns, and geographic distribution across multiple countries, with a specific focus on Africa.

**Methods:**

Three GIS dashboards were developed to address key objectives. The first integrates over 860,000 ATLAS data points from 83 countries, providing an interactive platform. Users can filter data by variables such as country, year, and region, enhancing data accessibility and visualization. The second dashboard focuses on the ATLAS dataset for Kenya and Uganda, incorporating detailed variables such as species, sample sources, and resistance phenotypes. The third involves Kampala, Uganda, to fill data gaps, enabling localized analyses through interactive features like geographic mapping and sample breakdowns by year.

**Results:**

Sub-Saharan Africa faces three major challenges in handling antimicrobial resistance (AMR) data: limited accessibility for non-technical users, inefficiencies in processing large datasets, and insufficient longitudinal data for analysis. The introduction of interactive dashboards significantly improved AMR data visualization and interpretation across different scales. The global AMR dashboard effectively mapped geographical trends, uncovering critical data gaps, particularly the scarcity of AMR records from Africa. The Kenya and Uganda dashboard revealed key resistance patterns, highlighting the ineffectiveness of Ceftriaxone, Erythromycin, Levofloxacin, and Ampicillin against E. coli isolates. Additionally, the Kampala-specific dashboard, developed using simulated data, demonstrated the potential for localized AMR visualization, providing valuable insights where real-world data is limited. Across all platforms, the dashboards' interactive features enhanced data accessibility and streamlined trend identification, making AMR insights more interpretable, especially for researchers in Sub-Saharan Africa.

**Conclusions:**

Interactive GIS dashboards enhance AMR data analysis in Sub-Saharan Africa by improving accessibility, efficiently handling large datasets, and addressing data gaps. Unlike spreadsheets such as Excel, which struggle with large datasets due to computer constraints, dashboards offer dynamic visualization, real-time updates, and intuitive data exploration.

## Plain Language Summary

Antimicrobial Resistance (AMR) is increasingly serious global health issue, making it harder to treat common infections due to the reduced effectiveness of antibiotics. To address this challenge, researchers, healthcare providers, and professionals need tools to better understand AMR. This study introduces an interactive tool in the form of Geographic Information System (GIS) dashboards, which allow users to explore and analyze AMR datasets. The first objective was to create a global map that tracks antibiotic usage and resistance trends across multiple countries, enabling users to zoom in on specific areas, such as countries or years, for more detailed insights from ATLAS dataset. The second objective was to develop maps for Kenya and Uganda, highlighting the unique AMR challenges faced in these countries. The third goal focused on using this tool to address data gaps in Kampala, Uganda, and help users analyze AMR trends. These interactive maps are highly valuable. The global map reveals trends and identifies areas where data is lacking, especially in Africa. The maps for Kenya and Uganda show concerning resistance levels, where antibiotics are becoming ineffective against bacteria like *E. coli*. The Kampala map demonstrates how the tool can help provide a more detailed analysis of local AMR issues. Overall, these maps make it easier for experts to explore AMR data and identify key trends. In simple terms, these GIS dashboards are powerful tools for communicating and raising awareness about AMR. They allow us to visualize the spread of resistance and explore detailed AMR data. These interactive dashboards could play a crucial role in the global effort to combat AMR.

## Introduction

AMR stands as a formidable global challenge, deeply intertwined with the framework of public health and healthcare systems across the world ^1^. Despite AMR's significant burden in sub-Saharan Africa, data on the region's AMR rates remain scarce ^2^. There is an urgent need to improve antibiotic stewardship and create context specific treatment guidelines, particularly in East Africa, where high rates of microbiologically confirmed urinary tract infections (UTIs) are driven largely by multidrug-resistant (MDR) Escherichia coli and Staphylococcus spp. ^3^. Research spotlighting antibiotic usage in hospitalized neonates reveals elevated mortality rates linked to culture-positive sepsis and notable antibiotic resistance ^4^. Diverse antibiotic regimens, often divergent from global guidelines, underscore the need for pragmatic trials to identify effective antibiotic protocols in regions grappling with substantial AMR challenges. Studies examining hurdles in implementing antimicrobial use (AMU) surveillance in Tanzania and Uganda highlight issues such as poor data quality, limited digitalization, and inadequate resources, underscoring the necessity for capacity building and continuous quality improvement ^5^. The evolution of national AMR surveillance systems in African countries from 2017 to 2019 is discussed, emphasizing constraints like limited laboratory capacity and staffing ^6^. A sustained increase in resistance among commonly used antibiotics in surgical wards of a Ugandan hospital from 2014 to 2018 underscores the need for ongoing monitoring, surveillance, and infection prevention and control practices ^7^.

Dashboards are powerful visual tools designed to consolidate and display diverse yet related data in one centralized location, making complex information easier to understand, analyze, and act upon ^8^. By integrating data from multiple sources, dashboards utilize visual elements such as graphs, charts, and tables to provide interactive overviews of key metrics and trends. These tools facilitate informed, data-driven decision-making by offering real-time updates and customizable views tailored to the needs of different users. Dashboards are employed across various industries, including healthcare, marketing, retail, agriculture, sales and other disciplines, where they help monitor performance, measure progress, and identify areas for improvement. They come in various types, such as strategic, analytics, KPI, project, and performance dashboards, each serving specific purposes such as tracking daily operations, visualizing high-level data, or analyzing key performance indicators. The benefits of dashboards include enhanced data clarity, centralized information access, real-time monitoring, and improved decision-making. Additionally, their intuitive design fosters better communication and collaboration ^8^. However, creating and maintaining dashboards requires technical expertise, consistent updates, and reliable data to ensure accuracy. Challenges such as high costs, information overload, and limited context can reduce their effectiveness if not carefully managed. Despite these challenges, dashboards remain essential tools for simplifying complex datasets and generating actionable insights ^8^.

Interactive Geographic Information System (GIS) dashboards integrate spatial data with traditional datasets to provide geographical context for analysis and decision-making in various sectors, including public health, disaster management, urban planning, and agriculture ^9^. GIS tasks span a wide range of processes, from data collection through techniques like GPS gathering and sensor integration, to data preprocessing, including image preparation and format standardization ^10, 11^. Data is often stored in solutions such as spatial data infrastructures and geodatabases, while spatial data mining techniques are employed to extract insights. Collaborative workflows are supported through multi-user geodatabases, while visualization tools facilitate the clear presentation of data via mapping and geo-visualization ^12^. Decision support tools, such as multi-criteria evaluation and suitability analysis, aid in strategic planning, while spatial analysis techniques like autocorrelation and hotspot analysis reveal patterns. Additional GIS functions include modeling, which simulates scenarios, and data dissemination through Web GIS, interactive maps, and geoportals, ensuring that spatial information is accessible and actionable ^9, 11, 12^.

Numerous studies underscore the critical role of dashboards in advancing AMR monitoring and stewardship. For example, the design of an AMR surveillance system tailored for neonatal intensive care units (NICUs) in Iran addresses inefficiencies in data analysis and reporting within antimicrobial stewardship programs. This system leverages principles of user experience design and business intelligence to ensure practical applicability ^13^. Similarly, integrating antibiotic stewardship indicators from electronic medical records (EMRs) highlights the potential of algorithmic data conversion and interactive dashboards to assess the appropriateness of antibiotic use, despite challenges like insufficient data quality and metadata categorization. These advancements emphasize the necessity of structured, interdisciplinary approaches to AMR surveillance to close critical data gaps ^14^. Kenya’s One Health AMR Surveillance System (OHAMRS) integrates human and animal health data to visualize AMR patterns across sectors ^15^, while Switzerland’s Swiss Pathogen Surveillance Platform (SPSP) leverages genomics and metadata for outbreak monitoring and data sharing ^16^. Globally, Merck’s SMART Surveillance program provides a robust dashboard tracking AMR trends across 83 countries, offering region-specific resistance insights ^17^. These initiatives underscore the significance of scalable and interoperable dashboards in enabling data-driven policies and fostering global collaboration. In Nepal ^18^, dashboard could be useful for streamlining AMR data management by providing real-time, dynamic visualization and analysis of standardized data, enabling evidence-based decision-making and efficient reporting for initiatives like WHO-GLASS ^19^. In Canada, The OHE-AMURS tool excelled by providing a structured, adaptable framework to evaluate AMR/AMU surveillance systems, synthesizing diverse knowledge sources, and using a transparent, repeatable matrix to assess 36 components across seven sustainability elements ^20, 21^. Its flexibility and robustness supported comprehensive evaluations of complex systems. However, further refinement is needed to better assess the scope of surveillance components and address coverage gaps.

Despite their transformative potential, AMR dashboards face significant challenges that can hinder their effectiveness. Insufficient data quality and gaps in metadata categorization remain persistent issues, limiting the accuracy and comprehensiveness of insights derived from these tools. For example, while integrating antibiotic stewardship indicators from EMRs can provide valuable data, the reliance on incomplete or poorly categorized metadata can undermine the reliability of algorithmic analyses. Additionally, the implementation of such systems often demands structured, interdisciplinary collaboration, which can be difficult to achieve due to varying expertise, resource availability, and sector-specific priorities. Scalability and interoperability, crucial for global initiatives like Kenya’s OHAMRS or Merck’s SMART Surveillance program, can also pose technical and logistical hurdles, especially in low-resource settings. Furthermore, the complexity of integrating diverse datasets from human, animal, and environmental health sectors, as seen in tools like the AR Dashboard, can lead to fragmented data visualization and hinder real-time decision-making. These limitations underscore the need for improved data infrastructure, enhanced training, and coordinated multi-sectoral efforts to maximize the potential of AMR dashboards.

The 2023 Vivli AMR Surveillance Data Challenge sought to advance antimicrobial resistance (AMR) research by encouraging the use of the Vivli AMR Register ^22^. This initiative enabled researchers to analyze surveillance data shared by GSK, Johnson and Johnson, Merck, Pfizer, Shionogi, Paratek, and Venatorx (with Merck's data excluded for the challenge). The goal was to detect resistance trends, inform policies, and model future scenarios. As part of this effort, we expressed interest in working with AMR data and submitted an abstract ^23^ for the Pfizer Antimicrobial Testing Leadership and Surveillance (ATLAS) dataset ^24, 25^.

### Objectives

**Figure 1.**
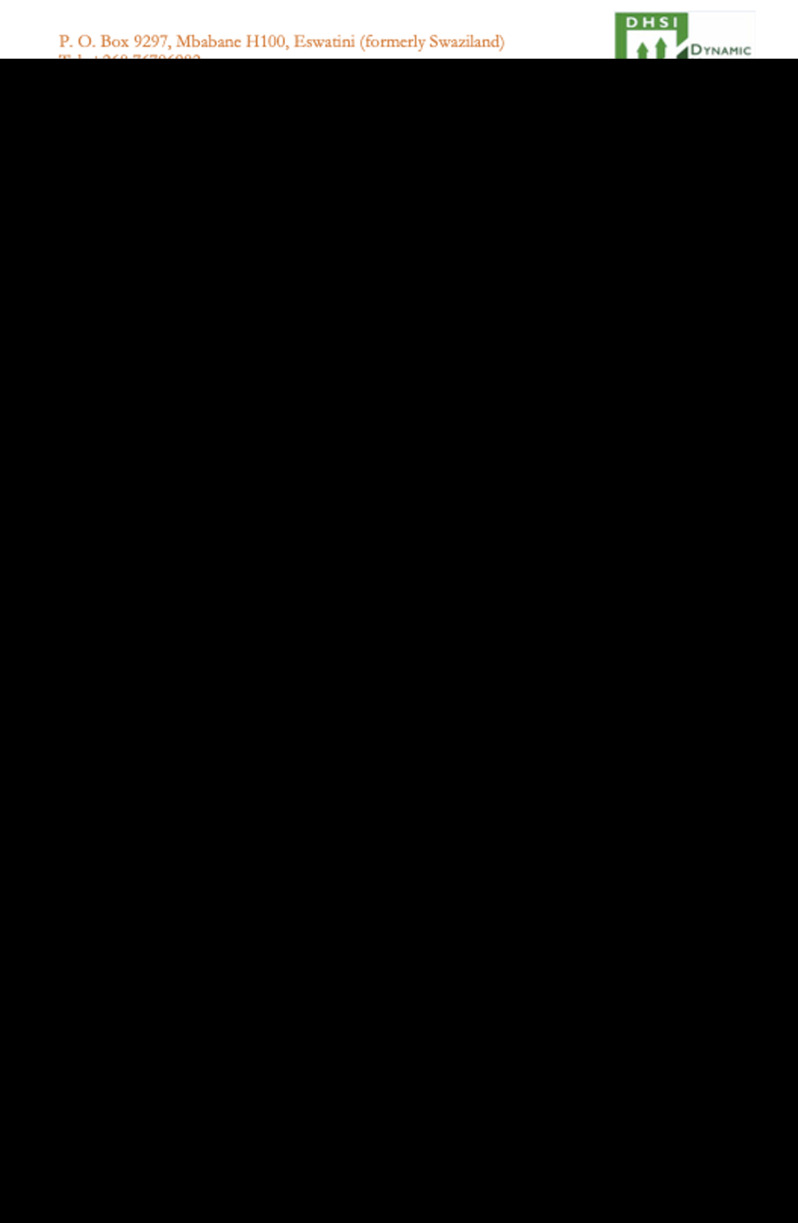
Barriers to AMR data analysis and visualization in Sub-Saharan Africa, including fragmented datasets, and limited computational infrastructure.

Upon receiving the dataset in a comma separated (CSV) format, we observed data gaps in African countries and that this motivated our three objectives. The dataset had limited data from African countries (13 out of 54 countries), making it difficult to generalize findings across the continent. Furthermore, the available data from African countries covered less than five years, reducing its usefulness for comprehensive analysis. Although we discovered a dataset from Uganda, it only contained information for 2021, while the dataset for Kenya spanned 2013, 2014, and 2021. Additionally, many researchers and public health officials in Sub-Saharan Africa face significant challenges in accessing and analyzing AMR data due to fragmented datasets, computational infrastructure limitations, and gaps in technical expertise ( Figure 1). The lack of high-performance computing resources in many institutions makes it difficult to process large datasets efficiently, while incomplete or poorly structured data further complicates analysis. Consequently, we adjusted our research approach to develop tools that address these challenges and beyond this competition. To fulfill this aim, we outlined the following objectives:

***Objective 1.*** Develop an interactive GIS dashboard using the Pfizer ATLAS dataset to complement spreadsheets. The dashboard will enhance AMR data exploration with real-time filtering and interactive visualization, making it more accessible and effective in resource limited settings.

***Objective 2.*** Create an interactive GIS dashboard specific to the Pfizer ATLAS data from Kenya and Uganda. This focused dashboard would provide a more detailed view of the data from these two countries.

***Objective 3.*** To demonstrate the potential usefulness and design of a dashboard that facilitates the analysis of antimicrobial resistance (AMR) patterns in Kampala, Uganda.

By accomplishing these objectives, we aimed to develop dashboards that would serve as valuable tools for researchers, and healthcare professionals using similar datasets. Ultimately, our goal is to provide a tool that could be utilized beyond the scope of this competition.

## Methods

### Pfizer ATLAS dataset

The ATLAS program, initiated by Pfizer in 2004, monitors global trends in antibiotic susceptibility, bacterial resistance, and the emergence of new resistance mechanisms across 83 countries ^24^. Local laboratories actively collect isolates, which are then sent to a central laboratory for standardized identification and susceptibility testing. This centralized approach ensures consistency in data quality. The program also incorporates genotypic data for selected beta-lactamase genes and antifungal susceptibility data through the SENTRY platform. Using Clinical and Laboratory Standards Institutes (CLSI) breakpoints with optional The European Committee on Antimicrobial Susceptibility Testing (EUCAST) analyses, ATLAS has tested over 850,000 antibiotic isolates and 21,000 antifungal isolates. Each record in the ATLAS dataset represents an individual patient and includes metadata such as the year and country of infection, the pathogen strain, and its classification as resistant, susceptible, or intermediate against a panel of antibiotics. The dataset also provides Minimum Inhibitory Concentrations (MICs) used for these classifications. Supplementary Figure 1 presents a screenshot displaying the initial rows and columns of the ATLAS dataset used in this study, which is available for download on GitHub at 26. The dataset contains 861,351 rows of isolates and 134 columns of variables shown in Supplementary Table 1, which can be downloaded from the GitHub at 27. The ATLAS dataset, derived from diverse surveillance programs, reflects considerable heterogeneity in its structure and content. Antibiotics tested for specific pathogens vary among patients, influenced by the unique requirements of surveillance programs or clinical contexts. Additionally, each MIC value is reported without replication or measures of variation, adhering to standard clinical practices. These factors contribute to significant spatial and temporal heterogeneities in antibiotic resistance patterns across various drug-bacterium pairs, as highlighted in the studies by 28, 29. Leveraging this dataset, which includes classifications of resistant, susceptible, and intermediate isolates, we developed Interactive GIS dashboards.

***Dataset for Objective 1.*** The dataset created for Objective 1 displays the distribution of isolates across 83 countries using a dashboard. It includes the following attributes: Isolate Id, Country, Year of data collection (sourced from the ATLAS dataset), and the geographical coordinates (longitude and latitude) along with the respective country regions, added to ATLAS dataset. The screenshot of the first few rows is visualized in Supplementary Figure 2, which can be downloaded from GitHub ^26^. The complete dataset is provided in Supplementary Table 2, also available for download on GitHub ^30^.

***Dataset for Objective 2.*** Objective 2 utilizes dataset from the ATLAS dataset, incorporating all 134 variables for Kenya and Uganda, along with the geographic coordinates (longitude and latitude) of Nairobi and Kampala, respectively. Detailed information on dataset is provided in Supplementary Table 3. The dataset can be accessed and downloaded via GitHub at 31.

***Dataset for Objective 3.*** For Objective 3, a dataset was simulated to demonstrate the functionality of the dashboard in Kampala, Uganda. This dataset is outlined in Supplementary Table 4 and is available for download on GitHub at 32.

### Statistical analysis

The ATLAS dataset underwent statistics analysis using the R statistical software version 4.2.2 ^33^.

### Chi-square test of proportions

A chi-square test of proportions was performed to examine the distribution of isolates across different regions, including Africa, Asia, Europe, Latin America and the Caribbean, Middle East, and North America. Additionally, another chi-square test was performed to assess the distribution of years of available number dataset across these regions.

### Identification of coordinates

To develop an interactive GIS dashboard, it is essential to have latitude and longitude positions for each country. To accomplish this, a coordinate was assigned to the capital city of each country, utilizing information from Wikipedia ^34^. These coordinates were then incorporated into the dataset, enabling accurate geographical representation and visualization within the dashboards.

## Results

***Statistical analysis.*** The dataset analyzed in this study consists of 83 countries and a total of 861,351 isolates. It encompasses 134 variables and includes information on 345 species. Table 1 provides an overview of the distribution of isolates across different regions and the availability of data over the years. The African region has the lowest number of datasets and the fewest years of available data, while Europe has the highest numbers. Chi-square tests confirmed that both the proportion of isolates in the region and the proportion of countries with at least 9 years of data significantly deviate from the expected proportion of 0.17 (p < 0.001, Table 2 and Table 3).

**Table 1.** Descriptive statistic results of the Atlas dataset as of June 15 ^th^, 2023: The dataset contains a total of 861,351 isolates. To determine the proportion, the number of isolates in each region is divided by the total number of isolates. Europe has the most extensive collection of datasets and the longest historical data, contrasting sharply with Africa, which possesses the least abundant dataset count and the shortest temporal coverage.

	Africa	Asia	Latin America and Caribbean	Middle East	Europe	North America
Number of isolates	22,908	117,213	96,566	32,129	420,714	171,821
Proportion of isolates per region	0.03	0.14	0.11	0.04	0.49	0.20

**Table 2.** The Atlas dataset, as of June 15th, 2023, shows the distribution of countries with 18 years of available data from 2004 to 2021. We set a criterion where each country should have at least 9 years of data out of the total 18-year period. The proportion was calculated by dividing the number of countries with at least 9 years of data by the total number of countries in each region. Isolates are unevenly distributed across various regions, such as Africa, Asia, Europe, Latin America, and the Caribbean, with Africa having only 13 representatives.

	Africa	Asia	Latin America and Caribbean	Middle East	Europe	North America
Number of countries	13	14	15	8	31	2
Number of countries with at least 9 years of dataset	2	11	8	4	23	2
Proportion of countries with at least 9 years of dataset	0.15	0.79	0.53	0.50	0.74	1.00

**Table 3.** Chi-square tests of proportions for the distribution of isolates across regions, and of distribution of countries with at least 9 years of dataset. The availability of dataset years varies among different regions, encompassing Africa, Asia, Europe, Latin America, and the Caribbean, with Africa exhibiting the shortest duration of available data.

	Chi-square	Df	p-value
Proportion of isolates per region	85.713	5	< 0.001
Proportion of countries with at least 9 years of dataset	69.717	5	< 0.001

## Development of web-based interactive GIS dashboards

The dashboards were developed using ArcGIS from Esri ^35^ and Shiny App ^36^.

**Figure 2.**
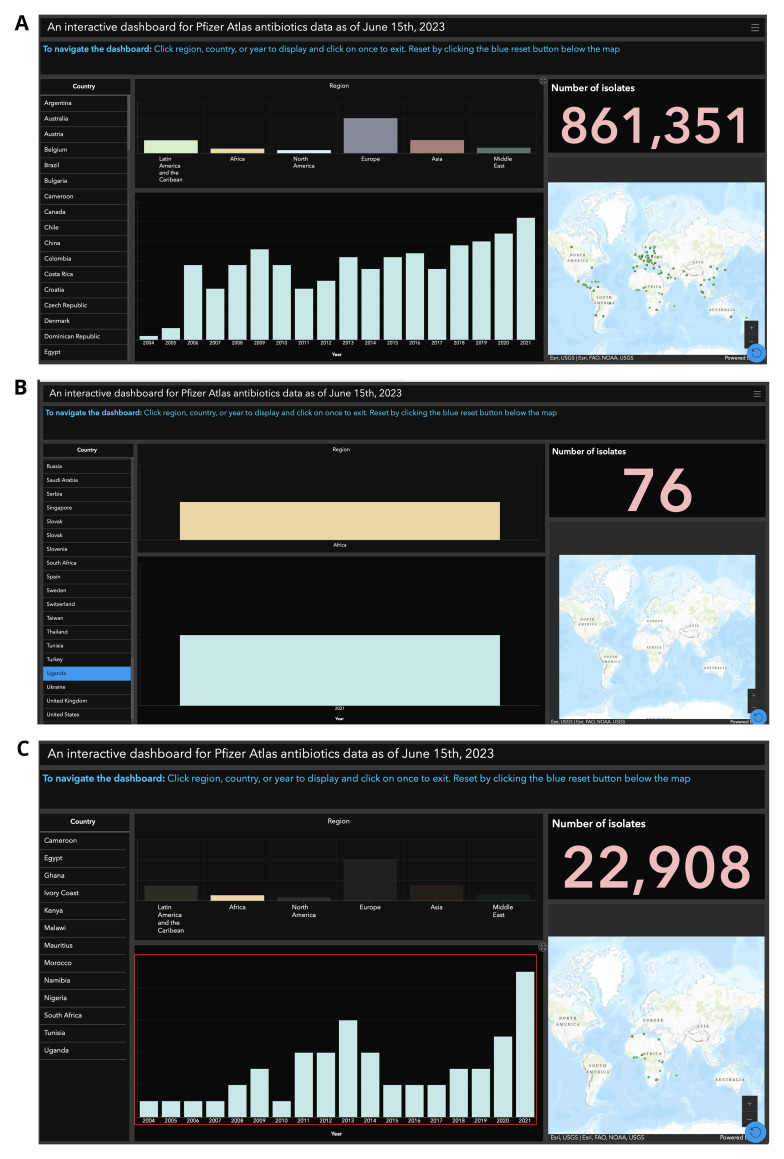
Interactive GIS Dashboards: **A**) Global Overview for 83 Countries, **B**) Uganda-Specific View, and **C**) Africa Regional Trends from 2004 to 2024. The global dashboard (Figure 2 **A**) features tools such as an interactive map, country selection, regional breakdowns, and intuitive navigation instructions, enabling users to analyze and explore data across multiple countries. In contrast, the Uganda-specific view (Figure 2 **B**) highlights data collected exclusively for 2021, with a total of 76 isolates, presented through a user-friendly interface designed for simplified data interpretation. Meanwhile, the Africa regional dashboard (Figure 2 **C**) illustrates the trend of data collection from 2004 to 2024, offering insights into regional patterns and temporal progress.

***Objective 1. Develop an interactive GIS dashboard using the Pfizer ATLAS dataset to complement spreadsheets. The dashboard will enhance AMR data exploration with real-time filtering and interactive visualization, making it more accessible and effective in resource limited settings.*** Displaying the dataset comprising over 860,000 rows from Supplementary Table 2, through an interactive GIS dashboard provides significant advantages over manual spreadsheet analysis, especially in resource limited setting such as Sub-Saharan Africa. In Sub-Saharan Africa, many institutions operate on low-specification computers that struggle with large datasets. Excel’s performance degrades significantly when handling AMR datasets with hundreds of thousands of rows. A dashboard offers immediate visual insights by organizing the data into a user-friendly format. For example, geographic mapping enables users to quickly identify patterns and trends across the 83 countries represented in the dataset. This spatial representation highlights areas with significant data gaps or regions requiring additional data collection, such as Africa, as shown in Figure 2A. Additionally, dashboards allow users to dynamically filter and segment data by country, year, For instance, a user can select a specific country, such as Uganda, to view detailed information like the 76 samples collected there in 2021 ( Figure 2B). This interactivity enables users to focus on the most relevant subsets of data, reducing manual effort and enhancing analysis efficiency.

Furthermore, visualizing sample numbers over time through bar charts or similar graphical formats highlights temporal trends in a way that raw spreadsheet data cannot. Patterns such as increases or decreases in sample collection become immediately evident, as illustrated in Figure 2C, which depicts data collected from Africa between 2004 and 2021. Lastly, integrating these elements into a cohesive, visually compelling dashboard interface makes it a powerful communication tool. Even users unfamiliar with complex datasets can intuitively interpret the information. The dashboard represented in Figure 2 can be accessed via a provided link ^37^.

***Objective 2. Create an interactive GIS dashboard specific to the ATLAS dataset from Kenya and Uganda.*** The specialized dashboard aims to provide a comprehensive and detailed perspective on ATLAS data from two specific nations of Kenya, and Uganda (Supplementary Table 3). The dataset comprises comprehensive details across several dimensions for AMR analysis. Each isolate is uniquely identified by an Isolate Id and linked to its source study (e.g., SPIDAAR). Biological and taxonomical data include the bacterial species (e.g., *Escherichia coli*, *Pseudomonas aeruginosa*) and their respective families. Geographical and demographic data provide location information (Country) and host demographics (Gender, Age Group). Clinical and contextual data capture details about the clinical specialty involved (e.g., Medicine General), the biological source of the sample (e.g., Urine, Wound), and whether the patient was classified as Inpatient or Outpatient. Temporal data document the year of sample collection. Resistance data consist of resistance phenotype classifications, MICs and Resistant, Susceptible, Intermediate, alongside specific resistance statuses for various antibiotics (e.g., Amikacin_I). Finally, the dataset includes genetic resistance markers such as CTXM1, KPC, and NDM, which indicate underlying resistance mechanisms. We added longitude and latitude for the capital city of both countries in the dataset.

However, this data structure has several disadvantages. Redundancy is a significant issue, as having separate columns for raw antibiotic measurements (e.g., MIC values) and interpretations can make the dataset unnecessarily large. Sparse data is also problematic, as many fields are incomplete, leading to substantial missing values. Furthermore, a lack of standardization in some categorical columns, such as specialty and source, increases variability and complicates analysis. The dataset's flat structure fails to capture complex relationships, such as those between isolates and resistance phenotypes, while geographic data lacks sufficient granularity for nuanced spatial analysis. The high dimensionality, with numerous columns for antibiotics, can lead to computational challenges. Additionally, overloaded columns like Amikacin, which mix qualitative data with quantitative MIC values, create difficulties in analysis, and ambiguous column labels or units hinder interpretation (Supplementary Table 3). Using an interactive GIS dashboard offers several advantages in addressing the challenges presented by the dataset. First, the dashboard enhances data visualization by transforming complex tabular data into intuitive and easily interpretable geographic maps, charts, and graphs. This makes it easier to identify spatial patterns, such antimicrobial resistance, and observe trends across locations and time. By integrating demographic, geographical, and resistance data on an interactive platform, the dashboard provides a unified view that reduces the redundancy and fragmentation inherent in the tabular format.

**Figure 3.**
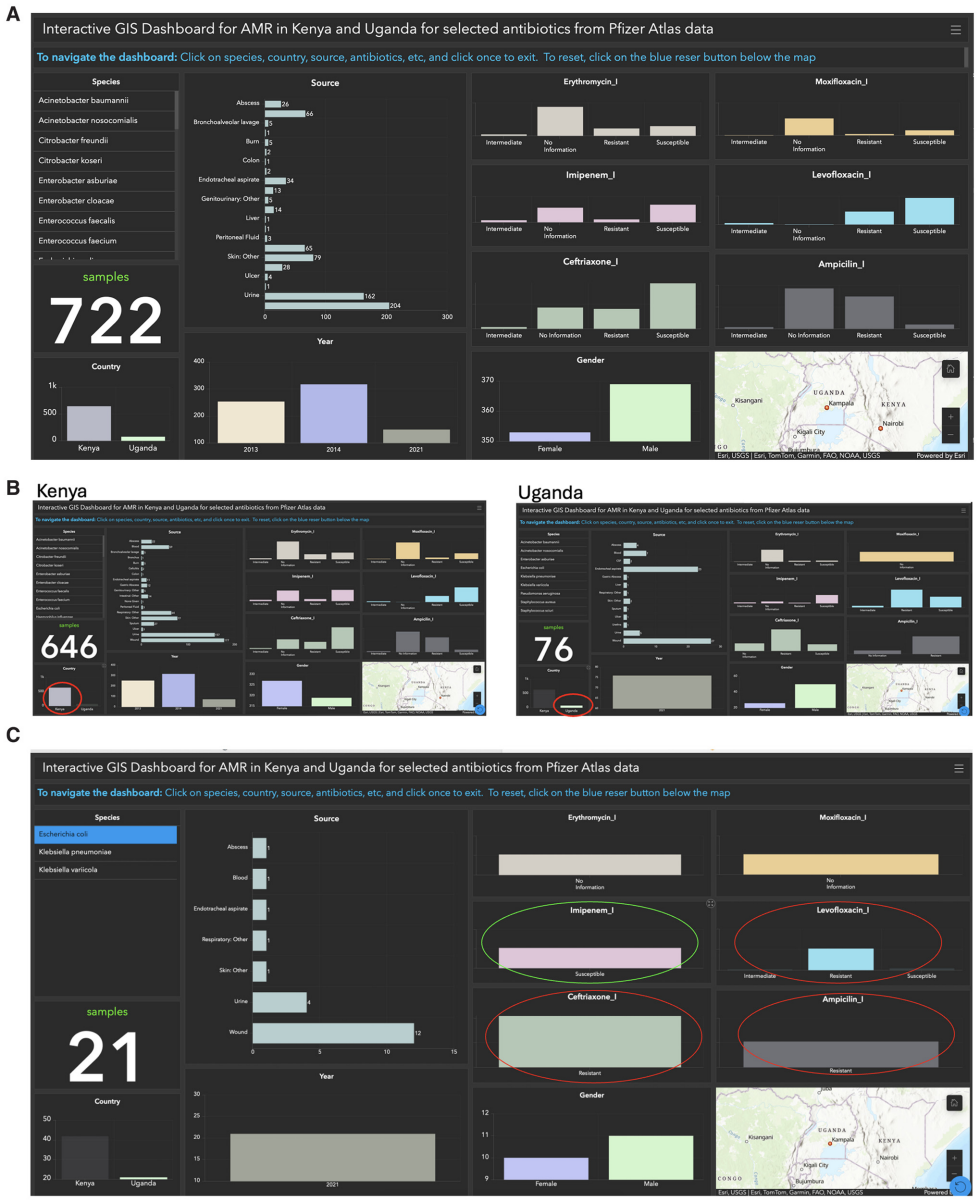
Interactive GIS Dashboards: The figure illustrates three components: **A**) a combined dashboard for Kenya and Uganda, highlighting key data points such as species, country, sample sources, counts, gender, and antibiotics; **B**) separate dashboards for Kenya and Uganda, offering country-specific insights; and **C**) antibiotic resistance in *E. coli* samples from Uganda, revealing resistance to Ampicillin, Levofloxacin, and Ciprofloxacin (red circles) in 21 samples, while all samples remained susceptible to Imipenem (green circle).

We developed an interactive GIS dashboard using the ATLAS dataset from Uganda and Kenya to demonstrate the advantages of streamlined data exploration and visualization. This dashboard focuses on key variables highlighted in red in Supplementary Table 3, including species, country, sample sources, sample counts, gender, and antibiotics ( Figure 3A). By providing an intuitive interface, the dashboard eliminates the need to manually search Supplementary Table 3 spreadsheet for specific data related to Uganda and Kenya ( Figure 3B). Users can apply filters based on these variables for targeted analysis, making the exploration of complex datasets efficient and user-friendly. To illustrate antibiotic resistance patterns in Uganda, we focused on *E. coli* isolate (highlighted in yellow in Supplementary Table 3) and six antibiotics in the columns: **Ampicillin_I**, **Ceftriaxone_I**, **Erythromycin_I**, **Imipenem_I**, **Levofloxacin_I**, and **Moxifloxacin_I** (highlighted in red). Analysis revealed that Ampicillin, Levofloxacin, and Ciprofloxacin were ineffective in 21 samples classified as Resistant, while Imipenem was effective across all samples, classified as Susceptible ( Figure 3C). Among the resistant samples, 12 originated from wounds, four from urine, and five from other sources ( Figure 3C).

This dashboard simplifies the visualization of antibiotic response data, offering a significant improvement over manually reviewing rows in Supplementary Table 3 for *E. coli* and comparing antibiotic resistance patterns. A similar interactive GIS dashboard can be adapted for use in countries where antibiotic test results are still recorded on paper, as highlighted in a recent study by The Mapping Antimicrobial Resistance and Antimicrobial Use Partnership (MAAP) ^38^. MAAP analyzed 819,584 AMR records collected between 2016 and 2019 from 205 laboratories across 14 countries: Burkina Faso, Cameroon, Eswatini, Gabon, Ghana, Kenya, Malawi, Nigeria, Senegal, Sierra Leone, Tanzania, Uganda, Zambia, and Zimbabwe. The study also included data from 327 hospital and community pharmacies and 16 national-level antimicrobial consumption (AMC) datasets, with some laboratory datasets being paper-based. By integrating such datasets into a digital, interactive GIS dashboard, these countries could significantly enhance their capacity to visualize and interpret AMR data. This tool would not only improve data accessibility but also empower laboratory technicians and researchers working with similar datasets.

The interactive GIS dashboard offers enhanced data exploration capabilities. Users can dynamically filter data based on criteria such as location, species, age group, or antibiotic type, enabling customized analyses without modifying the original dataset. This interactivity supports real-time comparisons and layered visualizations, uncovering relationships and correlations that may not be immediately evident in a spreadsheet format, such as that shown in Supplementary Table 3. Additionally, the dashboard provides a clear way to handle missing data by labeling it distinctly. For instance, missing values can be visualized as "No Information," as illustrated in Figures 3B and 3C for antibiotics, highlighting data gaps and guiding efforts to prioritize data collection. The dashboard can be accessed via a provided link ^39^.

**Figure 4.**
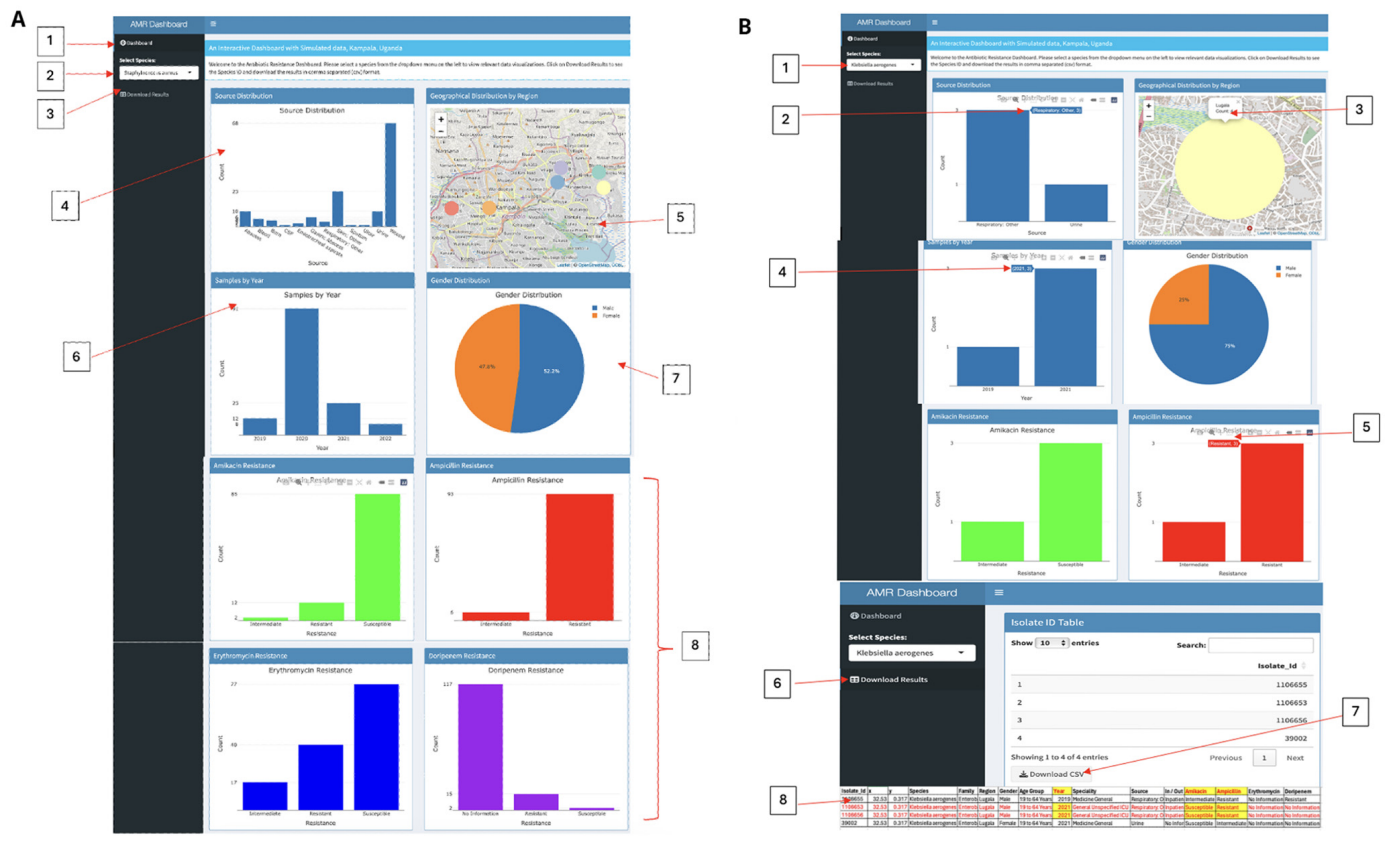
Interactive GIS dashboards: **A**) simulated Kampala data, **B**) Klebsiella aerogenes from only Lugala. Figure 4 **A** displays the simulated data examining various regions within Kampala, while Figure 4 **B** highlights the 2019 and 2021 detection of Klebsiella aerogenes exclusively in Lugala, revealing three resistance to Ampicillin but susceptibility to Amikacin. This simulated dataset, offers crucial insights into AMR trends and can be used to empower stakeholders for more informed decision-making.

***Objective 3. Demonstrate the functionality of the dashboard in Kampala, Uganda.*** As demonstrated in Supplementary Tables 2 and 3, as well as Figure 2B, Figure 3B and Figure 3C, Uganda had data available only for 2021. This suggests a potential lack of datasets specifically for Kampala, Uganda. To address this gap, we simulated a dataset modeled after ATLAS. Our simulated data focuses on analyzing various regions within Kampala, as illustrated in Figure 4. The dashboard [1], illustrated in, provides a comprehensive and interactive visualization of antibiotic resistance data using the R Shiny dashboard framework. At the top, a welcome message instructs: “Welcome to the Antibiotic Resistance Dashboard. Please select a species from the dropdown menu on the left to view relevant data visualizations. Click on 'Download Results' to access the Species ID and download the data in CSV format.” At the top left [2], users can choose from various species via a dropdown menu, with " *Staphylococcus aureus*" selected by default. Below this selection is the "Download Results" button [3], allowing users to export data in CSV format. The Source Distribution plot [4] showcases the count of samples across different sources such as "Abscess”, “Blood”,” Burn”, and others.

To the right of the source plot, a Geographical Distribution map [5] displays regional markers across Kampala, with interactive hover functionality revealing the name of each region and clickable markers that provide the total sample count for that region. Additional visualizations include the Samples by Year bar plot [6], which depicts the number of samples collected during the years 2019, 2020, 2021, and 2022. A Gender Distribution pie chart [7] illustrates the percentage of male and female participants, with a click feature revealing both the count and percentage values. Lastly, the Antibiotic Resistance plots [8] visualize resistance levels for antibiotics such as Amikacin (green) and Ampicillin (red), categorized into Intermediate, Resistant, Susceptible, or lacking information ^40^.

For illustration purpose, when " *Klebsiella aerogenes*" is selected from the species dropdown ( Figure 3B), the dashboard dynamically updates to display *K. aerogenes* visualizations. The Source Distribution Chart indicates that samples were collected from “Respiratory: Other” (3 samples) and “Urine” (1 sample). The Geographical Distribution Map pinpoints Lugala as the sole location of *K. aerogenes* cases, with a total of four cases reported. Additional insights are provided by the Samples by Year Plot, which shows an increase in sample counts over time: 1 sample in 2019, 1 sample in 2020, and 2 samples in 2021. The Antibiotic Resistance Charts reveal full resistance to Ampicillin (red, 4 cases) but full susceptibility to Amikacin (green, 4 cases). Users can export the filtered data using the "Download Results" button, which generates a CSV file. Notably, in 2021, K. aerogenes demonstrated resistance to Ampicillin (2 cases) while remaining susceptible to Amikacin (2 cases). Access the R Shiny dashboard via the provided link ^40^. It should be noted that this dashboard is solely for data visualization and presentation and does not perform any spatial analysis.

## Discussion

### The GIS-based platform vs. MS Excel and Tableau

In Sub-Saharan Africa, many institutions operate on low-specification computers that struggle with large datasets, gap in training, and lack of resources and limit the use of commonly used data analysis tools such as Microsoft Excel ^41^ and Tableau ^42^. While these tools offer basic data filtering and visualization functionalities, they are not fully equipped to handle the complexities of AMR data analysis in resource-constrained settings. Microsoft Excel, for instance, has severe computing constraints when dealing with large datasets, which is a critical issue in AMR surveillance where datasets often contain hundreds of thousands of records. Many public health institutions in Africa operate on low-specification computers, and Excel’s performance degrades significantly when processing such extensive data. Additionally, there is a skills gap among AMR researchers in Africa, as many lack advanced Excel training and rely primarily on basic functionalities. This reliance leads to inefficiencies in data extraction and analysis due to the need for repeated manual filtering and multi-step workflows. More importantly, Excel lacks geospatial analytics, limiting its ability to analyze AMR patterns based on geographical distribution, a crucial factor in understanding resistance trends across different regions.

Similarly, Tableau presents financial and accessibility barriers for AMR surveillance in Sub-Saharan Africa. Tableau is a commercial software that comes with high licensing costs, making it unaffordable for many institutions in low-income countries where AMR surveillance is urgently needed Tableau. Even though Tableau provides robust visualization tools, it does not inherently support the integration of multiple AMR surveillance sources, nor does it accommodate the inconsistent reporting structures often found in African datasets. Furthermore, effective use of Tableau requires specialized training, which poses an additional hurdle for public health professionals who may not have the resources or time to undergo such training Tableau. Given these limitations, our GIS-based platform offers a much-needed solution specifically designed to address the unique challenges of AMR data analysis in Sub-Saharan Africa. Unlike existing tools, our platform is built to bridge data gaps by integrating AMR data from multiple African sources, counteracting the underrepresentation of African AMR trends in global surveillance networks such as ATLAS, which currently includes data from only 13 out of 54 African countries, with limited years of data per country (ATLAS, 2023). Additionally, our dashboard is designed for usability, ensuring that non-technical users can access and interpret AMR data without requiring advanced data processing skills.

### Challenges in developing the interactive GIS dashboards

***Esri ArcGIS dashboards.*** The authors encountered multiple challenges while designing dashboards using ArcGIS dashboards, necessitating innovative solutions and adaptive strategies. One major hurdle was the platform's complexity and steep learning curve, which required users to possess a strong understanding of GIS concepts and Esri's tools. To address this, the team took a proactive approach to self-learning. They relied heavily on freely available online resources such as YouTube tutorials, which provided practical, step-by-step guidance on building and optimizing dashboards. This hands-on approach was complemented by extensive reading of official documentation, articles, and related materials, enabling the team to deepen their knowledge and effectively navigate the platform’s advanced features.

The challenges of handling large datasets were particularly pronounced, especially with the 645 MB ATLAS dataset with more than 860,000 rows and 134 columns. To optimize the dashboard's performance, the authors implemented several key strategies. For Objective 1, they reduced the dataset by selecting only three essential variables (Isolate Id, Country, and Year) out of the original 134 detailed in Supplementary Table 1. To enhance spatial analysis, they also incorporated geographic data such as regions and latitude and longitude. For Objective 2, they narrowed their focus to data from Uganda and Kenya, which not only reduced the dataset size but also allowed for a more targeted and manageable analysis. Additionally, the issue of missing data was addressed by labeling such entries as "No Information.", Figures 3A and 3B. This approach ensured completeness while improving interpretability for users, making the dashboards more user-friendly and informative. Another significant challenge was the high cost of ArcGIS dashboards, with Esri’s licensing model requiring on average $550 per user annually. Recognizing this financial burden, the team attempted to negotiate a reduced price with Esri. However, their efforts were unsuccessful due to the requirement for a larger number of user accounts to qualify for discounted rates. Despite this, the authors noted that such negotiations could be revisited in the future, especially for organizations with larger teams or those willing to explore collaborative licensing agreements.

By employing these solutions, streamlining variables, leveraging geographic data, targeting specific subsets of data, labeling missing values, investing in self-learning, and attempting cost negotiations, the authors successfully mitigated the challenges they faced. These adaptations not only optimized the dashboard’s performance but also improved its usability and accessibility, offering valuable lessons for others navigating similar obstacles. Their experience highlights the importance of creative problem solving, continuous learning, and strategic planning in the design and implementation of ArcGIS GIS dashboards.

***Shiny dashboard.*** The team also faced several challenges while developing a Shiny dashboard, which required creative solutions and a commitment to learning. One significant difficulty was the steep learning curve, as Shiny development demands not only proficiency in R programming but also familiarity with web technologies like HTML, CSS, and JavaScript. Advanced features further required a deep understanding of reactive programming and Shiny’s underlying infrastructure, making it challenging for those new to these areas. To build their expertise, the team relied on YouTube tutorials, which provided accessible, step-by-step guidance on Shiny development. This hands-on approach was supplemented with extensive reading of documentation and articles, enabling the team to enhance their skills and confidently navigate complex functionalities. Performance optimization was another hurdle, particularly creating intricate visualizations. Deployment and sharing also posed challenges, as hosting on platforms like ShinyApps.io or Shiny Server required careful management of server configurations, security protocols, and user access controls. Additionally, integrating the app with external systems or APIs required extra technical configuration. To address hosting concerns, the team set up their own Shiny server on DigitalOcean ^43^ providing more flexibility and control over the deployment process. This solution also proved cost-effective, with an average monthly expense of $30 for server maintenance, making it a sustainable choice compared to other hosting options.

***Impact of the work.*** The GIS web-based dashboards developed for the ATLAS dataset from the 83 countries, Kenya and Uganda, and the simulated dataset from Kampala provide essential tools for exploring and visualizing AMR data, especially in Sub-Saharan Africa. Many public health institutions in Sub-Saharan Africa face significant challenges in handling large AMR datasets due to computational constraints and skills gaps. Many researchers work with low-specification computers that struggle with large datasets, causing Excel’s performance to degrade when processing hundreds of thousands of rows. Additionally, the lack of advanced Excel training among AMR researchers results in inefficient workflows that rely on repetitive manual filtering and multi-step processes. These limitations hinder the rapid extraction of actionable insights in public health data. Addressing these challenges requires more efficient data processing tools and capacity-building initiatives to enhance analytical proficiency.

## Conclusion and limitations of the study

The development of interactive GIS dashboards using Esri ArcGIS and Shiny platforms offers tool for AMR visualization for Sub-Saharan Africa by allowing users to explore geographic and temporal trends in AMR data. However, several challenges were encountered during the development process. In the case of Esri ArcGIS dashboards, the platform's complexity and the steep learning curve presented significant hurdles, particularly for users with limited GIS experience. The large size of datasets, such as the ATLAS dataset, also posed problems, resulting in slow rendering and processing times, requiring optimization strategies like data aggregation. Additionally, the limited customization options and the high cost of Esri’s subscription model, create accessibility barriers for smaller organizations. Mobile optimization and managing user permissions also added complexity to the dashboard development process. Similarly, Shiny dashboards presented challenges, including the steep learning curve associated with R programming and web development. Deployment and sharing via platforms like ShinyApps.io also required managing server configurations and user access, presenting challenges for those without advanced web development skills. Integrating Shiny with external systems or APIs further increased the complexity of the development process. The study also highlights the scarcity and inconsistencies in ATLAS AMR data, particularly in Africa, where limited sample sizes and variability in data collection across regions like Uganda and Kenya reduce the reliability and generalizability of the dashboards. The dashboards, therefore, depend on standardized data collection practices, improved laboratory capabilities, and greater investment in healthcare infrastructure to fully realize their potential in addressing AMR in Africa.

## Data Availability

The ATLAS dataset cannot be made available as it is under license by a third party. However, the dataset can be requested from Vivli ^44^ using the following information:- Data contributor name: Pfizer (AMR); DOI: https://doi.org/10.25934/PR00007930; AMR ID: VIV00007930; and Dataset ID: ATLAS_Antibiotics The dataset for Objective 1, Supplementary_Table_2.xlsx is available for download at GitHub in the following ^30^, similarly, dataset for Objective 2, Supplementary_Table_3.xlsx.zip ^31^, is available at GitHub at 30, and dataset for Objective 3, Supplementary_Table_4.xlsx, is available at GitHub ^32^. This publication is based on research using data from Pﬁzer, obtained through ^44^. The dashboard creation codes for Objectives 1 and 2 are not available, as the dashboards were originally constructed using the graphical user interface (GUI) on ArcGIS. To replicate the dashboards, we can leverage open-source QGIS GIS software ( https://qgis.org/en/site/), utilizing the information outlined in the materials and methods section. The R Shiny app call “app.R” for creating dashboard for Objective 3 is available at GitHub in 45, and information about the Shiny app and instructions for installing the app is available in RMarkdown format, pdf format, and html format at GitHub in 46.
