# Supplementary material for: Empowering Global AMR Research Community: Interactive GIS dashboards for AMR data analysis and informed decision-making
Source: Wellcome Open Res. 2025 Mar 7;9:234. Originally published 2024 May 2. [Version 4] doi: 10.12688/wellcomeopenres.21010.4 (PMC11910713; doi:10.12688/wellcomeopenres.21010.4)
Supplement: Peer review history

# Reviewer report: Reviewer response for version 4

**DOI:** 10.21956/wellcomeopenres.26371.r120300

**Published:** 2025-03-15

**Authors:** Simon Reid

**Affiliations:** 1 The University of Queensland, Herston, Queensland, Australia

The authors have addressed my comments well. I think the paper makes a worthy contribution to the field.

Is the work clearly and accurately presented and does it cite the current literature?

Partly

If applicable, is the statistical analysis and its interpretation appropriate?

Yes

Are all the source data underlying the results available to ensure full reproducibility?

Yes

Is the study design appropriate and is the work technically sound?

Partly

Are the conclusions drawn adequately supported by the results?

No

Are sufficient details of methods and analysis provided to allow replication by others?

Yes

Reviewer Expertise:

I have epidemiological expertise in animal and public health, with a specific emphasis on the analysis, design and use of surveillance data/systems. I have a particular interest in the systems and processes associated with decision-making in public health.

I confirm that I have read this submission and believe that I have an appropriate level of expertise to confirm that it is of an acceptable scientific standard.

# Reviewer report: Reviewer response for version 4

**DOI:** 10.21956/wellcomeopenres.26371.r120298

**Published:** 2025-03-12

**Authors:** Liam P Shaw

**Affiliations:** 1 University of Oxford, Oxford, England, UK

Thanks to the authors for their responses and revisions. I think the way the study is presented now avoids misrepresenting the work undertaken. I also think that the authors' conclusions and assessments are now more clearly presented in the restructured results and discussion.

Is the work clearly and accurately presented and does it cite the current literature?

Partly

If applicable, is the statistical analysis and its interpretation appropriate?

Partly

Are all the source data underlying the results available to ensure full reproducibility?

Partly

Is the study design appropriate and is the work technically sound?

Partly

Are the conclusions drawn adequately supported by the results?

No

Are sufficient details of methods and analysis provided to allow replication by others?

No

Reviewer Expertise:

I am a computational biologist. Previously I have written my own article arising from a data reuse challenge on AMR (https://wellcomeopenresearch.org/articles/4-86) and also peer reviewed another article from a data reuse challenge authored by Quentin Leclerc and others (https://wellcomeopenresearch.org/articles/4-140).

I confirm that I have read this submission and believe that I have an appropriate level of expertise to confirm that it is of an acceptable scientific standard.

# Reviewer report: Reviewer response for version 3

**DOI:** 10.21956/wellcomeopenres.26004.r117159

**Published:** 2025-02-25

**Authors:** Liam P Shaw

**Affiliations:** 1 University of Oxford, Oxford, England, UK

I have read the article again. The authors have made positive changes, such as citing previous work on AMR dashboards in their introduction, including a new supplementary table, and including descriptions of the work they did. I am sorry to say that I cannot approve the study in its current state. 

 This is no comment on the work, but because the authors still do not seem to have taken onboard the comments of the peer reviewers about their description of the study being inaccurate. For example, in the abstract they still state that they were "simplifying data interpretation for diverse users and supporting informed decision-making processes", which implies (to me) that the study somehow looked at decision-making processes. But it didn't. 

 Reading their most recent responses, I think that this is just a fundamental disagreement between the authors and their reviewers. For example, the authors write in their response to my review that their dashboard "significantly improv[es] the user experience by eliminating the need for tedious scrolling and searching through the ATLAS dataset, which contains over 860,000 rows of isolates and 134 columns of variables." But this is arguably a strawman since the ATLAS dataset is, for many people, available via an existing dashboard https://atlas-surveillance.com/. And as Simon Reid's most recent review points out, many of these features are easily available in spreadsheets: even if downloaded as a spreadsheet then "scrolling and searching" is not required - one can just use a filter on a column. 

 I commend the authors on their work. But they seem committed to the way they have presented their results when different reviewers have expressed similar concerns. That is their prerogative. The advantage of a platform like Wellcome Open Research is that other people can read their article as it is and make up their own minds.

Is the work clearly and accurately presented and does it cite the current literature?

Partly

If applicable, is the statistical analysis and its interpretation appropriate?

Partly

Are all the source data underlying the results available to ensure full reproducibility?

Partly

Is the study design appropriate and is the work technically sound?

Partly

Are the conclusions drawn adequately supported by the results?

No

Are sufficient details of methods and analysis provided to allow replication by others?

No

Reviewer Expertise:

I am a computational biologist. Previously I have written my own article arising from a data reuse challenge on AMR (https://wellcomeopenresearch.org/articles/4-86) and also peer reviewed another article from a data reuse challenge authored by Quentin Leclerc and others (https://wellcomeopenresearch.org/articles/4-140).

I confirm that I have read this submission and believe that I have an appropriate level of expertise to state that I do not consider it to be of an acceptable scientific standard, for reasons outlined above.

# Response

**Published:** 2025-02-27

**Authors:** Stephen Opiyo

**Affiliations:** Patira Data Science, LLC, Westerville, Ohio, USA

I have read the article again. The authors have made positive changes, such as citing previous work on AMR dashboards in their introduction, including a new supplementary table, and including descriptions of the work they did. I am sorry to say that I cannot approve the study in its current state.

**Reviewer's comments** This is no comment on the work, but because the authors still do not seem to have taken onboard the comments of the peer reviewers about their description of the study being inaccurate. For example, in the abstract they still state that they were "simplifying data interpretation for diverse users and supporting informed decision-making processes", which implies (to me) that the study somehow looked at decision-making processes. But it didn't.

**Response to Reviewer** Based on your suggestion, we have revised the results section of the abstract to enhance clarity and conciseness. Specifically, we have removed the phrase "simplifying data interpretation for diverse users and supporting informed decision-making processes” as requested while ensuring the key findings remain effectively conveyed. The updated results section now reads as follows: *Sub-Saharan Africa faces three major challenges in handling antimicrobial resistance (AMR) data: limited accessibility for non-technical users, inefficiencies in processing large datasets, and insufficient longitudinal data for analysis. The introduction of interactive dashboards significantly improved AMR data visualization and interpretation across different scales. The global AMR dashboard effectively mapped geographical trends, uncovering critical data gaps, particularly the scarcity of AMR records from Africa. The Kenya and Uganda dashboard revealed key resistance patterns, highlighting the ineffectiveness of Ceftriaxone, Erythromycin, Levofloxacin, and Ampicillin against E. coli isolates. Additionally, the Kampala-specific dashboard, developed using simulated data, demonstrated the potential for localized AMR visualization, providing valuable insights where real-world data is limited. Across all platforms, the dashboards' interactive features enhanced data accessibility and streamlined trend identification, making AMR insights more interpretable, especially for researchers in Sub-Saharan Africa.*

**Reviewer’s comments** Reading their most recent responses, I think that this is just a fundamental disagreement between the authors and their reviewers. For example, the authors write in their response to my review that their dashboard "significantly improv[es] the user experience by eliminating the need for tedious scrolling and searching through the ATLAS dataset, which contains over 860,000 rows of isolates and 134 columns of variables." But this is arguably a strawman since the ATLAS dataset is, for many people, available via an existing dashboard https://atlas-surveillance.com/. And as Simon Reid's most recent review points out, many of these features are easily available in spreadsheets: even if downloaded as a spreadsheet then "scrolling and searching" is not required - one can just use a filter on a column.

**Response to Reviewer**

**Addressing the perceived "Strawman" Argument** We acknowledge the fundamental disagreement between our position and the reviewers' perspective on the necessity and added value of our dashboards. While we appreciate the points raised, we maintain that our approach offers distinct advantages over existing Excel-based workflows and the ATLAS dashboard, particularly in the context of AMR data usability in Sub-Saharan Africa. Our response clarifies these distinctions and highlights the practical challenges faced by researchers in low-resource settings. Some of the authors are from Africa, and our approach is grounded in the realities of data accessibility, computational infrastructure, and public health needs in the region. **Limited African representation in ATLAS**

- ATLAS includes data from only 13 out of 55 African countries, leaving out critical regions where AMR surveillance is needed.
- Some of these 13 countries have only two years of data, significantly limiting the ability to analyze trends and develop meaningful interventions.
- In contrast, countries like the UK have AMR data dating back to 2004, providing a longitudinal dataset for deeper epidemiological analysis.
- This data gap disadvantages African researchers and policymakers, forcing reliance on fragmented data or global datasets that do not accurately represent local resistance patterns.

**Excel’s limitations in sub-Saharan Africa**

- **Computing constraints and large dataset handling:** Many institutions operate on low-specification computers that struggle with large datasets. Excel’s performance degrades significantly when handling AMR datasets with hundreds of thousands of rows.
- **Skills and training gaps:** Many AMR researchers in African public health institutions lack advanced Excel training and rely on basic functionalities. The need for repeated manual filtering and multi-step workflows creates inefficiencies, making it difficult for public health professionals to quickly extract actionable insights.

**Why a custom dashboard is necessary** Our dashboard is not just an alternative to Excel filtering, it is a purpose built solution designed to overcome critical limitations faced by researchers in Sub-Saharan Africa.

1. **Bridging data gaps**: ATLAS lacks data from most African countries. Our dashboard is specifically designed to integrate and visualize African-specific AMR data, addressing this gap and providing a more comprehensive regional perspective.
2. **Enhancing usability for non-technical users** – Unlike Excel, which requires manual filtering and technical expertise, our dashboard features an intuitive interface, making data analysis accessible to researchers without advanced data skills.
3. **Optimizing performance** – Large AMR datasets often overwhelm Excel, causing performance issues. Our dashboard efficiently processes complex queries, ensuring smooth analysis without the need for high-end computing resources.
4. **Addressing financial constraints** – Many research institutions in Sub-Saharan Africa lack the resources to purchase expensive third-party software like Tableau. Our dashboard provides a cost-effective, open-source alternative without financial barriers.

 
**Reviewer’s comments** I commend the authors on their work. But they seem committed to the way they have presented their results when different reviewers have expressed similar concerns. That is their prerogative. The advantage of a platform like Wellcome Open Research is that other people can read their article as it is and make up their own minds. **Response to Reviewer** We sincerely appreciate the reviewer’s thoughtful feedback and acknowledge the differences in perspective regarding the necessity and value of our dashboards. While we recognize and respect the concerns raised, we maintain that our approach offers significant advantages over existing Excel-based workflows and the ATLAS dashboard, particularly in the context of AMR data usability in Sub-Saharan Africa. Our response aims to clarify these distinctions and highlight the practical challenges that researchers in low-resource settings face. Some of our authors are from Africa, and our work is directly informed by the realities of data accessibility, computational infrastructure, and public health needs in the region.

**Final thoughts** We value the open peer-review process and appreciate the opportunity to clarify our position. The advantage of publishing on platforms like Wellcome Open Research is that readers can critically evaluate our work and form their own conclusions. While we acknowledge the differences in opinion, we remain confident that our approach provides a practical and necessary solution for AMR data analysis in African contexts. Once again, we thank the reviewers for their insights, which have helped us furth

# Reviewer report: Reviewer response for version 3

**DOI:** 10.21956/wellcomeopenres.26004.r117544

**Published:** 2025-01-27

**Authors:** Simon Reid

**Affiliations:** 1 The University of Queensland, Herston, Queensland, Australia

I agree with other reviewers that this paper presents an interesting and well-developed tool, and that my comments are associated mainly with the presentation of the paper and the interpretation of the results.

 The structure of the paper is somewhat confusing because there is no major heading for the results section and no clear discussion section. There appears to be a paragraph of results under the italicised "Statistical analysis" section that is under a sub-heading called "Identification of coordinates". It is not clear what that result presents other than the measurement of variation in the data coverage between regions. 

 The fundamental problem I have is that the first two objectives present a discussion of the benefits of the GIS-based platform that only uses a single geographic coordinate based on the capital city. Many of the benefits presented also exist in simple databases such as MS Excel, which has simple and easy to use filtering capabilities, plus visualisation tools in the form of pivot charts and tables that allow exploration, visualisation and some fairly reasonable data analysis. There are also fairly simple data visualisation tools that are commonly used for surveillance dashboards using Tableau, for example. The discussion presented doesn't seek to compare these similar tools with the GIS-based visualisation tool. 

 I appreciate that at a higher spatial resolution (Objective 3) there are some benefits in the display of data. However, the study does not perform any spatial analysis only data visualisation. I think the authors need to be clear that the tool does not enable analysis beyond that achievable with simple pivot tables and charts in MS Excel. 

 The study would benefit from some information from the perspective of the "decision-makers" who are assumed to need the dashboard. This is necessary before claims of benefits are made.

Is the work clearly and accurately presented and does it cite the current literature?

Partly

If applicable, is the statistical analysis and its interpretation appropriate?

Yes

Are all the source data underlying the results available to ensure full reproducibility?

Yes

Is the study design appropriate and is the work technically sound?

Partly

Are the conclusions drawn adequately supported by the results?

No

Are sufficient details of methods and analysis provided to allow replication by others?

Yes

Reviewer Expertise:

I have epidemiological expertise in animal and public health, with a specific emphasis on the analysis, design and use of surveillance data/systems. I have a particular interest in the systems and processes associated with decision-making in public health.

I confirm that I have read this submission and believe that I have an appropriate level of expertise to state that I do not consider it to be of an acceptable scientific standard, for reasons outlined above.

# Response

**Published:** 2025-02-27

**Authors:** Stephen Opiyo

**Affiliations:** Patira Data Science, LLC, Westerville, Ohio, USA

Reviwer's comments I agree with other reviewers that this paper presents an interesting and well-developed tool, and that my comments are associated mainly with the presentation of the paper and the interpretation of the results.

 The structure of the paper is somewhat confusing because there is no major heading for the results section and no clear discussion section. There appears to be a paragraph of results under the italicised "Statistical analysis" section that is under a sub-heading called "Identification of coordinates". It is not clear what that result presents other than the measurement of variation in the data coverage between regions.   

**Response to Reviewer** Thank you for your insightful feedback. We have made substantial revisions to the manuscript to improve clarity and address the structural concerns. Specifically, we have:

1. **Clearly separated the Results and Discussion sections** to enhance the logical flow of the paper..
2. **Expanded on the challenges of AMR surveillance in Sub-Saharan Africa** and the need for an alternative dashboard to address these gaps.

**Addressing the reviewer’s specific concerns** **1. Structure of the paper – Results and Discussion separation** We acknowledge that the lack of a major heading for the Results section may have contributed to structural ambiguity. In the revised version, we have clearly delineated the Results and Discussion sections.

- The **Results section** now presents an explicit analysis of data distribution across regions, highlighting the disparities in AMR data representation in Sub-Saharan Africa compared to high-income countries.
- The **Discussion section** contextualizes these findings, addressing the limitations of existing datasets (e.g., ATLAS) and the challenges posed by using Excel for AMR surveillance in low-resource settings.

**2. Results presentation – ATLAS Dataset and African representation** We have clarified the statistical results related to data representation:

- Over the last **18 years**, ATLAS has maintained **comprehensive** AMR data collection for developed nations, whereas Sub-Saharan African data remains fragmented and inconsistent.
- ATLAS includes only **13 out of 55 African countries**, leaving large gaps in AMR surveillance across the continent.
- Even within these **13 African countries**, data collection is sporadic. For example:
  - **Egypt:** Data was collected only from **2011 to 2013**.
  - **Nigeria:** Data was available only for **2013–2014 and 2018–2021**, with significant gaps between **2004–2012 and 2015–2017**.
  - **In contrast**, the **United Kingdom has data spanning 2004 to 2021**, allowing for longitudinal epidemiological analysis.
- Of the **800,000+ isolates** in ATLAS, only **22,000 isolates** originated from Africa, demonstrating the scale of underrepresentation.

These findings underscore why AMR research in Africa is severely constrained by incomplete datasets, necessitating alternative.

**Reviewer’s Comments**

 The fundamental problem I have is that the first two objectives present a discussion of the benefits of the GIS-based platform that only uses a single geographic coordinate based on the capital city. Many of the benefits presented also exist in simple databases such as MS Excel, which has simple and easy to use filtering capabilities, plus visualisation tools in the form of pivot charts and tables that allow exploration, visualisation and some fairly reasonable data analysis. There are also fairly simple data visualisation tools that are commonly used for surveillance dashboards using Tableau, for example. The discussion presented doesn't seek to compare these similar tools with the GIS-based visualisation tool. 

**Response to Reviewer** We appreciate the reviewer’s feedback and recognize the importance of clarifying the distinctions between our GIS-based platform and existing data visualization tools such as MS Excel and Tableau. Our response clarifies these differences and highlights the practical challenges faced by researchers in low-resource settings. Some of the authors are from Africa, and our approach is grounded in the realities of data accessibility, computational infrastructure, and public health needs in the region. **1. The GIS-based platform vs. MS Excel and Tableau** We acknowledge that tools such as **Excel and Tableau** provide data filtering and visualization capabilities; however, they do not fully address the core challenges faced in AMR data analysis in **Sub-Saharan Africa**.

- **Excel’s Limitations in Sub-Saharan Africa:**
  - **Computing constraints and large dataset handling:** Many African institutions operate on low-specification computers that struggle with large AMR datasets. Excel’s performance significantly degrades when handling datasets with hundreds of thousands of rows.
  - **Skills and training gaps:** Many AMR researchers in African public health institutions lack advanced Excel training, relying on basic functionalities. The need for repeated manual filtering and multi-step workflows makes it difficult to extract actionable insights efficiently.
  - **Lack of geographic context:** Excel lacks geospatial analytics, limiting the ability to analyze AMR trends based on geographical distribution.
- **Tableau’s financial and accessibility barriers:**
  - **High Cost:** Tableau is an expensive commercial software, making it inaccessible to many institutions in **low-income countries** where AMR surveillance is most needed.
  - **Data integration issues:** While Tableau provides visualization tools, it does not inherently integrate multiple AMR surveillance sources or accommodate the inconsistent reporting structures present in African datasets.
  - **Technical expertise requirement:** Unlike our **custom-built dashboard**, Tableau requires training to use effectively, which may not be feasible for all public health professionals.

**2. Why our GIS-based platform is necessary** Our dashboard is not simply an alternative to existing tools but a **purpose-built solution** designed to overcome the limitations faced by researchers in Sub-Saharan Africa:

- **Bridging data gaps:** ATLAS includes data from only **13 out of 55 African countries**, with some of these countries having only **two years of data**. Our dashboard **integrates African-specific AMR data**, addressing the **underrepresentation** of the region and providing a **comprehensive regional perspective**.
- **Enhancing usability for non-technical users:** Unlike **Excel or Tableau**, which require manual filtering and technical expertise, our dashboard **features an intuitive interface**, making data visualization **accessible to researchers without advanced data skills**.
- **Optimizing performance:** AMR datasets are **large and complex**, often exceeding **Excel’s computational capacity**. Our **dashboard efficiently processes complex queries**, ensuring visualization and presentation **without requiring high-end computing resources**.
- **Addressing financial constraints:** Many institutions **lack the budget to purchase** expensive **third-party software** like Tableau. Our dashboard provides a **cost-effective, open-source alternative**, eliminating **financial barriers** for public health institutions in Africa.

**3. Why a GIS-based approach is important** Unlike standard database filtering, a GIS-based approach provides **geospatial insights** that are critical for AMR surveillance. While some benefits of data filtering exist in Excel or Tableau, our platform provides:

- **Mapping resistance trends** across regions, allowing targeted interventions.
- **Identifying geographic hotspots** for AMR, which **Excel filtering cannot achieve**.
- **Real-time surveillance capabilities**, which go beyond **static spreadsheet analysis**.

**Final adjustments to the manuscript** In response to this feedback, we have updated the discussion section to **compare our GIS-based tool with Excel and Tableau**, explicitly stating the **advantages of our approach** in an African context. These revisions ensure that the **contribution of our platform is clearly articulated** while addressing the reviewer’s concerns about alternative tools.

**Reviewer’s Comments** I appreciate that at a higher spatial resolution (Objective 3) there are some benefits in the display of data. However, the study does not perform any spatial analysis only data visualisation. I think the authors need to be clear that the tool does not enable analysis beyond that achievable with simple pivot tables and charts in MS Excel.   

**Response to Reviewer**

 We have indicated in the text that the tool does not perform spatial analysis.

**Reviewer’s Comments** The study would benefit from some information from the perspective of the "decision-makers" who are assumed to need the dashboard. This is necessary before claims of benefits are made.  

**Response to Reviewer** We appreciate the reviewer’s insightful comment regarding the need for perspectives from decision-makers who are assumed to need the AMR dashboard before making claims about its benefits. We recognize the importance of stakeholder engagement in ensuring that the dashboard is relevant and valuable to those involved in AMR surveillance and decision-making. To this end, we have obtained a formal letter from a key stakeholder, Mr. Sylvester Z. Moyo, Director of Programs at Dynamic Health Systems Initiative (https://github.com/so13839/Team-8952-Vivli-AMR-Competation/blob/main/data/Letter%20of%20Appreciation_AMR%20Dashboard.pdf). In his letter, Mr. Moyo articulates the challenges faced by AMR professionals in Africa, particularly the limitations of existing data management tools like Excel and the high costs of proprietary software such as Tableau and Esri. He highlights the practical benefits of our AMR dashboard, including its ability to handle large datasets, generate real-time visualizations, and provide an accessible, open-source alternative tailored to the needs of African stakeholders. His endorsement underscores the relevance of this dashboard in addressing AMR challenges in resource-limited settings. Furthermore, several of the authors of this study are based in Africa and have direct interactions with stakeholders who recognize the value of dashboards in overcoming data management barriers. Given the widespread AMR challenges across the continent, stakeholders frequently emphasize the need for efficient, cost-effective data visualization tools to support evidence-based decision-making. These interactions have informed the development of the dashboard, ensuring that it meets real-world needs and enhances AMR data accessibility and usability. Based on this input, we believe that the AMR dashboard presents a meaningful solution that aligns with the needs of decision-makers and researchers working on AMR in Africa. We will continue to engage with stakeholders to refine and expand the dashboard’s functionality to maximize its impact. We appreciate the reviewer’s feedback and remain committed to ensuring that our study is informed by stakeholder perspectives.

# Reviewer report: Reviewer response for version 2

**DOI:** 10.21956/wellcomeopenres.25363.r112467

**Published:** 2024-12-13

**Authors:** Liam P Shaw

**Affiliations:** 1 University of Oxford, Oxford, England, UK

**Disclaimer:** I have read the other referee's reports before writing my own.

 Thanks to the authors for the article and I appreciate the work they did in putting this dashboard together. Unfortunately I have major concerns. I know this can be frustrating but I make these comments in the spirit of constructive feedback. I apologise for any oversights or misunderstandings. 

 To summarise, I agree with the serious criticisms raised by Quentin Leclerc in his review. The main problem is that the presentation of the study, particularly in the abstract, is inaccurate: the authors claim that "the study demonstrates that the developed GIS dashboards empower stakeholders". However, it doesn't do this. The same applies to many other statements in the paper. This is not a criticism of the work the authors did do, but of how they have presented it. The authors should accurately describe their work rather than appearing to claim they have done something else. 

 I have some minor comments on the various objectives below which relate to how the work itself was performed.

**Previous literature**

 The authors do not cite any previous dashboards in their introduction. But these exist e.g. SMART from Merck. Others can be found by e.g. searching "dashboard antibiotic resistance" on PubMed (15 results). 

**Objective 1:**

 The global dashboard ( https://patira.maps.arcgis.com/apps/dashboards/d89c5b039552464f8f04103f05ac889f) has some features that confuse me. What are the green dots in the map on the right-hand side? I thought these were indicating countries, but if e.g. I zoom in on the UK I see two green dots within its borders. Then if I click on Ireland, there is no green dot on the map within Ireland. The same problem arises for Belgium. I think these are probably quite mundane bugs but they are quite serious if the aim of the dashboard is to display data accurately. It doesn't give me confidence in the dashboard, and raises the question of to what extent Objective 1 has been achieved. I presume these errors arise from the longitude/latitude for capital cities obtained from Wikipedia but since these weren't made available and I'm not sure of the precise source I cannot check (the Wikipedia citation is only for the Wikipedia homepage). 

 Regarding the visualization for an individual country e.g. Uganda in Fig. 1b: the authors claim that this is "a visually appealing presentation...simplifying data interpretation, aiding monitoring and evaluation efforts, and providing comprehensive information for hypothesis formulation, particularly emphasizing the examination of the number of isolates and data collection duration in the country". But this confuses me, because these two pieces of information are the only ones available in the display: there are 76 isolates and they were collected in 2021. As far as I'm aware the dashboard presents no other information. Since there isn't any other data to interpret, this is not "comprehensive information for hypothesis formulation". It's hard for me to reconcile what the authors have done with how it has been described. 

**Objective 2:**

 When I try to reproduce Fig 2b by clicking "Escherichia coli" and "Uganda" I get 26 samples rather than 21. I was confused about this inconsistency. Then when I clicked "Resistant" for levofloxacin I could get 21 samples. I think the authors should describe that this is the filtering they did: their figure shows only the *E. coli *samples from Uganda that were resistant to levofloxacin, which allows a user to see resistance levels for other antibiotics.  

**Objective 3:**

 I don't understand why generating a simulated dataset is the objective. Making a dashboard for hypothetical data at the country level is surely the objective? The simulated data is arbitrary. The link (reference 20) didn't work for me and then I realised that I would need to use the github to reproduce it - that should be made clear rather than saying it's accessible at the link, since it isn't without those steps. 

 In summary, unfortunately it is not possible for me to endorse this work given these concerns.

Is the work clearly and accurately presented and does it cite the current literature?

Partly

If applicable, is the statistical analysis and its interpretation appropriate?

Partly

Are all the source data underlying the results available to ensure full reproducibility?

Partly

Is the study design appropriate and is the work technically sound?

Partly

Are the conclusions drawn adequately supported by the results?

No

Are sufficient details of methods and analysis provided to allow replication by others?

No

Reviewer Expertise:

I am a computational biologist working. I have written my own article arising from a data reuse challenge on AMR (https://wellcomeopenresearch.org/articles/4-86) and also peer reviewed another article from a data reuse challenge authored by Quentin Leclerc and others (https://wellcomeopenresearch.org/articles/4-140).

I confirm that I have read this submission and believe that I have an appropriate level of expertise to state that I do not consider it to be of an acceptable scientific standard, for reasons outlined above.

# Response

**Published:** 2024-12-25

**Authors:** Stephen Opiyo

**Affiliations:** Patira Data Science, LLC, Westerville, Ohio, USA

Dear Dr. Liam P. Shaw, Thank you very much for taking the time to review our manuscript. We sincerely appreciate your valuable feedback. We will respond to each of your comments thoroughly. 

 Kind regards, 

 Stephen  

**Reviewer comments** **Disclaimer:** I have read the other referee's reports before writing my own.

 Thanks to the authors for the article and I appreciate the work they did in putting this dashboard together. Unfortunately I have major concerns. I know this can be frustrating but I make these comments in the spirit of constructive feedback. I apologise for any oversights or misunderstandings. 

 To summarise, I agree with the serious criticisms raised by Quentin Leclerc in his review. The main problem is that the presentation of the study, particularly in the abstract, is inaccurate: the authors claim that "the study demonstrates that the developed GIS dashboards empower stakeholders". However, it doesn't do this. The same applies to many other statements in the paper. This is not a criticism of the work the authors did do, but of how they have presented it. The authors should accurately describe their work rather than appearing to claim they have done something else. 

**Response to the Reviewer** The abstract has been rewritten to reflect the results discussed in the revised manuscript (see Page 1). Additionally, I have addressed the questions and comments raised by Quentin Leclerc. 

**Below are questions and comments raised by Quentin Leclerc and our responses** **Reviewer comments** In this article, the authors present GIS dashboards they designed using data from industry AMR monitoring systems. Such tools can be interesting to visualise AMR data, both for communication and awareness purposes. The design of the dashboards by the authors is appropriate and I have no major concerns about this. Unfortunately, the conclusions of the study are currently too strong and not supported by the analysis. While I agree that GIS dashboard can be useful, here the authors do not directly demonstrate that they empower stakeholders or allow key decisions to be made. In that sense, the “Impact of the work” section of the Discussion is misleading as it discusses the potential usefulness of dashboard as if this was a certainty that had been proven in this article. Additionally, I think that further information should be provided on the datasets used in this project. Overall, I believe this article can be of general interest only if the conclusions are rephrased to appropriately discuss the potential usefulness of GIS dashboards as well as their limitations. Additionally, I think that the paper would be greatly enhanced by the authors’ perspective on designing such a dashboard: which obstacles did they encounter? How have they adapted their approach? Do they have any recommendations? These questions would be of interest to the scientific community. If such changes are implemented, I would be happy to update my review. **Response to the Reviewer**

*Datasets and dashboards* We have clarified and expanded on the datasets used for developing the dashboards: Supplementary Table 2: Dataset for the Objective 1 dashboard. Supplementary Table 3: Dataset for dashboards developed for Objective 2. Supplementary Table 4: Dataset for creating the Objective 3 dashboard. These datasets have been made publicly available for transparency and reproducibility via GitHub at https://github.com/so13839/Team-8952-Vivli-AMR-Competation/tree/main/data. References 30 to 32 in the revised manuscript now correspond to these datasets. *Impact of the Work* We have revised the “Impact of the Work” section to provide greater emphasis on the dashboards' usability and significance:   The GIS web-based dashboards developed for the ATLAS dataset, covering 83 countries, and the dashboards for Kenya, Uganda, and simulated data from Kampala provide essential tools for exploring and analyzing AMR data. These dashboards are designed to offer a user-friendly, interactive interface tailored to researchers, technicians, and healthcare professionals. The dashboards allow filtering and segmenting of data by key variables, such as country, species, sample sources, and antibiotic resistance profiles. For instance, the Uganda and Kenya dashboards highlight resistance patterns for *E. coli* and visualize data distributions by sample source, year, and resistance levels for antibiotics such as Ampicillin, Levofloxacin, and Imipenem. In Kampala, the simulated dataset provides a focused view, enabling users to identify areas with limited data and prioritize research. Missing data is clearly labeled as “No Information,” helping to highlight gaps requiring further investigation. This interactive approach offers significant improvements over traditional spreadsheet analysis by uncovering spatial and temporal trends efficiently and enabling informed decision-making. *Conclusions and Limitations of the Study* We have rephrased the conclusions to emphasize the dashboards’ potential while addressing their limitations: The interactive GIS dashboards developed using Esri ArcGIS and Shiny represent a significant improvement over spreadsheets, enabling efficient exploration of geographic and temporal trends in AMR data. However, several challenges were encountered. For Esri ArcGIS dashboards, these included the platform's complexity, steep learning curve, and the large dataset size, which required optimization strategies such as data aggregation to improve performance. Limited customization options and the high subscription costs of Esri tools also posed barriers, especially for smaller organizations. Shiny dashboards presented challenges such as the steep learning curve of R programming, deployment complexities, and integration with external APIs. Managing server configurations for platforms like ShinyApps.io required advanced technical skills. Additionally, the study highlights the limitations in AMR data availability, particularly in Africa. Data scarcity, limited sample sizes, and inconsistencies in data collection practices across Uganda and Kenya reduce the dashboards’ reliability. To address these challenges and enhance the dashboards’ effectiveness, we recommend standardized data collection practices, investment in laboratory infrastructure. These steps will ensure the dashboards’ full potential is realized in combating AMR in Africa.

**Reviewer comments**

**Introduction**

 The Introduction can be refocused on the topic of the study – for example, although I agree they are important, the concepts of One Health and antibiotic consumption in animals are not directly relevant in this work. Instead, it would be more relevant for you to introduce GIS dashboards in greater detail – why did the OHE-AMURS work so well? Are there any limitations? How could dashboards be useful in Nepal? By focusing on GIS dashboards before the Objectives section, the scope of the article will be much clearer for readers. **Response to the Reviewer** We have revised the manuscript by removing references to One Health and antibiotic consumption in animals. Instead, we have introduced Geographic Information Systems (GIS) and an Interactive GIS Dashboard as central components of the study. Additionally, we have addressed all the reviewer’s comments in the Introduction, specifically within paragraphs 2 to 5 of the revised manuscript.

**Reviewer comments** **Objectives**

 It is not clear here what you mean by “descriptive statistical analysis”. If this refers to the beginning of your Results section, it is a bit hard to follow, as it explains the conclusions before the results. I understand you may be describing your exact work process during the Vivli Data Challenge, but for readers this will not be clear. You could simply state here that you observed data gaps in African countries and that this motivated your three objectives.

 Throughout: you used the ATLAS dataset in your analysis, so please correct any references to the data accordingly. For example, “the Vivli dataset” is not accurate, since Vivli only facilitate access to the data, but do not own it.

 Objective 3 needs to be clarified. It is not obvious what you mean by “simulated dataset” here, how this may be useful, and what is the link with Kampala, Uganda.  

**Response to the Reviewer** We have removed the descriptive statistical analysis and highlighted the observation of data gaps in African countries in the revised manuscript. Additionally, the Vivli dataset has been replaced with the ATLAS dataset. Objective 3 has been rewritten as: 'To demonstrate the potential usefulness and design of a dashboard that facilitates the analysis of antimicrobial resistance (AMR) patterns in Kampala, Uganda.'"

**Reviewer comments**

**Methods**

 Further information needs to be provided on the ATLAS dataset which you used to inform the dashboards, otherwise readers cannot understand the context and generalisability of the work. For example: how were isolates collected, what is the purpose of this data, which variables does it contain, which biases could it be exposed to... This has been discussed in previous articles which I have referenced below.

**Response to the Reviewer** We have incorporated a discussion of the ATLAS dataset in the revised manuscript, detailing the methods used for isolate collection and referencing previous studies that analyzed this data. Please refer to the section titled Pfizer ATLAS Dataset on page 4 of the revised manuscript.

**Reviewer comments**

**Results**

 Here already, you should adjust the phrasing on the usefulness of GIS dashboards. Here, since you do not directly demonstrate the usefulness of dashboards, you can only discuss their potential value. For example, “is a valuable tool for researchers, policymakers and healthcare professionals” is not supported by the analysis.

**Response to the Reviewer** We have carefully revised the section as per your feedback. The updated section can now be found on page 6 of the revised manuscript. This revision incorporates the necessary clarifications and aligns with the overarching goals of the study. We believe these changes enhance the manuscript's clarity and relevance. If additional adjustments or further clarifications are required, we are happy to address them.  

**Reviewer comments**

 In Objective 2, it is not clear what you mean by “comparative analysis and focused investigations”.  

**Response to the Reviewer** We have carefully addressed your comment and have rewritten and clarified the content in Objective 2, which can now be found on page 6 of the revised manuscript.

**Reviewer comments** For Objective 3 here again it is very unclear what the “simulated dataset” corresponds to and how it was generated. If it is simulated, then interpreting susceptibility results for *K. aerogenes* does not make sense. If the point here is to demonstrate the potential usefulness and design of a dashboard using this type of data, then it should be presented as such.  

**Response to the Reviewer** We have revised the objective to clarify that the simulated dataset is intended to demonstrate how the dashboard could be applied to Kampala, Uganda. The Shiny dashboard created using this simulated data is for illustrative purposes only. The primary intention is to showcase the functionality and potential application of the dashboard. We have included this clarification in the revised manuscript, which can be found on page 7.

**Reviewer comments**

**Discussion**

 A major counter-argument to the use of GIS dashboards is that researchers interested in in-depth analysis would simply obtain the underlying data themselves, rather than use the dashboard. In that sense, you should better discuss the usefulness of dashboards: to whom and how would they be useful exactly? Do you have any examples of dashboards being used in analyses? Are there any previous studies which discussed the potential benefits of GIS dashboards for AMR surveillance? (or for any other surveillance)  

**Response to the Reviewer** In response to your suggestion, we have emphasized the advantages of using dashboards over spreadsheets, particularly in the context of Objectives 1 to 3. Dashboards offer enhanced data visualization, real-time updates, and more intuitive decision-making compared to traditional spreadsheet approaches. For example, Kenya’s One Health AMR Surveillance System (OHAMRS) integrates human and animal health data to visualize AMR patterns across various sectors, making it easier to detect and respond to emerging threats. Similarly, Switzerland’s Swiss Pathogen Surveillance Platform (SPSP) leverages genomics and metadata for effective outbreak monitoring and data sharing. These case studies demonstrate the value of dashboards in surveillance and their ability to improve the efficiency and accessibility of complex data sets. References 15 and 16 in the revised manuscript have been updated to reflect these examples.

**Reviewer comments** As mentioned previously, there are several statements in your Discussion which are not supported by your analysis, such as “The dashboard acts as a centralized hub, aggregating and organizing these datasets for easy access and analysis.” which is not the case here since (if I understood correctly) you only used the ATLAS dataset; “complex queries” are not demonstrated since you present only filtering commands; “examine the impact of different policies or interventions in specific countries, evaluate the effectiveness of existing strategies” policies and interventions are not implemented in the dashboard; “healthcare outcomes” are not included in the dashboard.  

**Response to the Reviewer** We have revised the **Impact of the Work** and **Conclusions sections** to better reflect the findings of our study. The updated content can be found on pages 9 to 10 of the revised manuscript. We believe these revisions provide a clearer and more accurate representation of the implications of our results.  

**Response to Dr. Liam P. Shaw continued**

 I have some minor comments on the various objectives below which relate to how the work itself was performed.

**Reviewer comments** **Previous literature**

 The authors do not cite any previous dashboards in their introduction. But these exist e.g. SMART from Merck. Others can be found by e.g. searching "dashboard antibiotic resistance" on PubMed (15 results). 

**Response to the Reviewer** We have included additional literature in the Introduction section of the revised manuscript, specifically in paragraph 4, page 3. This new content references previous work on dashboards related to Antimicrobial Resistance (AMR), as indicated by references 13 to 21. These references discuss the development and application of AMR dashboards, providing insights into their role in data visualization, surveillance, and decision-making.

**Reviewer comments** **Objective 1:**

 The global dashboard ( https://patira.maps.arcgis.com/apps/dashboards/d89c5b039552464f8f04103f05ac889f) has some features that confuse me. What are the green dots in the map on the right-hand side? I thought these were indicating countries, but if e.g. I zoom in on the UK I see two green dots within its borders. Then if I click on Ireland, there is no green dot on the map within Ireland. The same problem arises for Belgium. I think these are probably quite mundane bugs but they are quite serious if the aim of the dashboard is to display data accurately. It doesn't give me confidence in the dashboard, and raises the question of to what extent Objective 1 has been achieved. I presume these errors arise from the longitude/latitude for capital cities obtained from Wikipedia but since these weren't made available and I'm not sure of the precise source I cannot check (the Wikipedia citation is only for the Wikipedia homepage). 

**Response to the Reviewer** The green dots indicate countries. We have corrected the error on the map, and now when you click on the green dot representing a country, the name of the country is displayed. The longitude and latitude used to create the dashboard are provided in Supplementary Table 2 for reference.

**Reviewer comments**

 Regarding the visualization for an individual country e.g. Uganda in Fig. 1b: the authors claim that this is "a visually appealing presentation...simplifying data interpretation, aiding monitoring and evaluation efforts, and providing comprehensive information for hypothesis formulation, particularly emphasizing the examination of the number of isolates and data collection duration in the country". But this confuses me, because these two pieces of information are the only ones available in the display: there are 76 isolates and they were collected in 2021. As far as I'm aware the dashboard presents no other information. Since there isn't any other data to interpret, this is not "comprehensive information for hypothesis formulation". It's hard for me to reconcile what the authors have done with how it has been described.   

**Response to the Reviewer** The dashboard is designed to allow users to dynamically filter Uganda from the 83 countries in the ATLAS dataset, significantly improving the user experience by eliminating the need for tedious scrolling and searching through the ATLAS dataset, which contains over 860,000 rows of isolates and 134 columns of variables.

**Reviewer comments**

**Objective 2:**

 When I try to reproduce Fig 2b by clicking "Escherichia coli" and "Uganda" I get 26 samples rather than 21. I was confused about this inconsistency. Then when I clicked "Resistant" for levofloxacin I could get 21 samples. I think the authors should describe that this is the filtering they did: their figure shows only the *E. coli *samples from Uganda that were resistant to levofloxacin, which allows a user to see resistance levels for other antibiotics.  

**Response to the Reviewer** In response to your comment, we have revised the manuscript to clarify the filtering process used in our analysis. Specifically, the figure now highlights only the *E. coli* samples from Uganda, revealing that Ampicillin, Levofloxacin, and Ciprofloxacin were ineffective in 21 samples, which were classified as Resistant. Conversely, Imipenem was effective across all 21 samples, which were classified as Susceptible. This updated explanation is provided in Figure 2B on page 7 of the revised manuscript.

**Reviewer comments** **Objective 3:**

 I don't understand why generating a simulated dataset is the objective. Making a dashboard for hypothetical data at the country level is surely the objective? The simulated data is arbitrary. The link (reference 20) didn't work for me and then I realised that I would need to use the github to reproduce it - that should be made clear rather than saying it's accessible at the link, since it isn't without those steps. 

**Response to the Reviewer** We have revised Objective 3 to better reflect the purpose of the Objective. Initially, the objective aimed to demonstrate the functionality of the dashboard in Kampala, Uganda, specifically addressing the challenges posed by the limited availability of AMR datasets. To overcome this, we simulated a dataset to ensure the dashboard’s functionality could still be showcased. However, following your suggestion, we have reworded the objective to focus on demonstrating the functionality of the interactive GIS dashboard itself. This revision highlights the dashboard's capabilities in visualizing and analyzing antimicrobial resistance (AMR) data, regardless of dataset limitations, by leveraging both real and simulated data. We believe this approach more accurately conveys the core aim of the study.

# Reviewer report: Reviewer response for version 2

**DOI:** 10.21956/wellcomeopenres.25363.r111333

**Published:** 2024-12-07

**Authors:** Quentin Leclerc

**Affiliations:** 1 Conservatoire national des arts et metiers (Ringgold ID: 27054), Paris, Île-de-France, France

In this article, the authors present GIS dashboards they designed using data from industry AMR monitoring systems. Such tools can be interesting to visualise AMR data, both for communication and awareness purposes. The design of the dashboards by the authors is appropriate and I have no major concerns about this. Unfortunately, the conclusions of the study are currently too strong and not supported by the analysis. While I agree that GIS dashboard can be useful, here the authors do not directly demonstrate that they empower stakeholders, or allow key decisions to be made. In that sense, the “Impact of the work” section of the Discussion is misleading as it discusses the potential usefulness of dashboard as if this was a certainty that had been proven in this article. Additionally, I think that further information should be provided on the datasets used in this project. Overall, I believe this article can be of general interest only if the conclusions are rephrased to appropriately discuss the potential usefulness of GIS dashboards as well as their limitations. Additionally, I think that the paper would be greatly enhanced by the authors’ perspective on designing such a dashboard: which obstacles did they encounter? How have they adapted their approach? Do they have any recommendations? These questions would be of interest to the scientific community. If such changes are implemented, I would be happy to update my review. My comments addressed to the authors are listed below.

**Introduction**

 The Introduction can be refocused on the topic of the study – for example, although I agree they are important, the concepts of One Health and antibiotic consumption in animals are not directly relevant in this work. Instead, it would be more relevant for you to introduce GIS dashboards in greater detail – why did the OHE-AMURS work so well? Are there any limitations? How could dashboards be useful in Nepal? By focusing on GIS dashboards before the Objectives section, the scope of the article will be much clearer for readers.

**Objectives**

 It is not clear here what you mean by “descriptive statistical analysis”. If this refers to the beginning of your Results section, it is a bit hard to follow, as it explains the conclusions before the results. I understand you may be describing your exact work process during the Vivli Data Challenge, but for readers this will not be clear. You could simply state here that you observed data gaps in African countries and that this motivated your three objectives.

 Throughout: you used the ATLAS dataset in your analysis, so please correct any references to the data accordingly. For example, “the Vivli dataset” is not accurate, since Vivli only facilitate access to the data, but do not own it.

 Objective 3 needs to be clarified. It is not obvious what you mean by “simulated dataset” here, how this may be useful, and what is the link with Kampala, Uganda.

**Methods**

 Further information needs to be provided on the ATLAS dataset which you used to inform the dashboards, otherwise readers cannot understand the context and generalisability of the work. For example: how were isolates collected, what is the purpose of this data, which variables does it contain, which biases could it be exposed to... This has been discussed in previous articles which I have referenced below.

**Results**

 Here already, you should adjust the phrasing on the usefulness of GIS dashboards. Here, since you do not directly demonstrate the usefulness of dashboards, you can only discuss their potential value. For example, “is a valuable tool for researchers, policymakers and healthcare professionals” is not supported by the analysis.

 In Objective 2, it is not clear what you mean by “comparative analysis and focused investigations”.

 For Objective 3 here again it is very unclear what the “simulated dataset” corresponds to and how it was generated. If it is simulated, then interpreting susceptibility results for *K. aerogenes* does not make sense. If the point here is to demonstrate the potential usefulness and design of a dashboard using this type of data, then it should be presented as such.

**Discussion**

 A major counter-argument to the use of GIS dashboards is that researchers interested in in-depth analysis would simply obtain the underlying data themselves, rather than use the dashboard. In that sense, you should better discuss the usefulness of dashboards: to whom and how would they be useful exactly? Do you have any examples of dashboards being used in analyses? Are there any previous studies which discussed the potential benefits of GIS dashboards for AMR surveillance? (or for any other surveillance)

 As mentioned previously, there are several statements in your Discussion which are not supported by your analysis, such as “The dashboard acts as a centralized hub, aggregating and organizing these datasets for easy access and analysis.” which is not the case here since (if I understood correctly) you only used the ATLAS dataset; “complex queries” are not demonstrated since you present only filtering commands; “examine the impact of different policies or interventions in specific countries, evaluate the effectiveness of existing strategies” policies and interventions are not implemented in the dashboard; “healthcare outcomes” are not included in the dashboard.

Is the work clearly and accurately presented and does it cite the current literature?

Yes

If applicable, is the statistical analysis and its interpretation appropriate?

Yes

Are all the source data underlying the results available to ensure full reproducibility?

Partly

Is the study design appropriate and is the work technically sound?

Partly

Are the conclusions drawn adequately supported by the results?

No

Are sufficient details of methods and analysis provided to allow replication by others?

No

Reviewer Expertise:

My research is focused on the dynamics of AMR. I have previously worked with industry AMR monitoring systems, including the ATLAS dataset used in this study (see https://wellcomeopenresearch.org/articles/4-140 and https://wellcomeopenresearch.org/articles/9-248). I won three prizes in the context of data challenges involving these datasets (2019, 2023, 2024).

I confirm that I have read this submission and believe that I have an appropriate level of expertise to state that I do not consider it to be of an acceptable scientific standard, for reasons outlined above.

## References

[1] : Seeking patterns of antibiotic resistance in ATLAS, an open, raw MIC database with patient metadata. *Nat Commun* .2022;13(1) : 10.1038/s41467-022-30635-7 2917 10.1038/s41467-022-30635-7 35614098 PMC9133080

[2] : Determinants of worldwide antibiotic resistance dynamics across drug-bacterium pairs: a multivariable spatial-temporal analysis using ATLAS. *Lancet Planet Health* .2023;7(7) : 10.1016/S2542-5196(23)00127-4 e547-e557 10.1016/S2542-5196(23)00127-4 37437996

# Response

**Published:** 2024-12-25

**Authors:** Stephen Opiyo

**Affiliations:** Patira Data Science, LLC, Westerville, Ohio, USA

Dear Dr. Quentin Leclerc, Thank you very much for taking the time to review our manuscript. We sincerely appreciate your valuable feedback. We will respond to each of your comments thoroughly. 

 Kind regards, 

 Stephen  

**Reviewer comments** In this article, the authors present GIS dashboards they designed using data from industry AMR monitoring systems. Such tools can be interesting to visualise AMR data, both for communication and awareness purposes. The design of the dashboards by the authors is appropriate and I have no major concerns about this. Unfortunately, the conclusions of the study are currently too strong and not supported by the analysis. While I agree that GIS dashboard can be useful, here the authors do not directly demonstrate that they empower stakeholders or allow key decisions to be made. In that sense, the “Impact of the work” section of the Discussion is misleading as it discusses the potential usefulness of dashboard as if this was a certainty that had been proven in this article. Additionally, I think that further information should be provided on the datasets used in this project. Overall, I believe this article can be of general interest only if the conclusions are rephrased to appropriately discuss the potential usefulness of GIS dashboards as well as their limitations. Additionally, I think that the paper would be greatly enhanced by the authors’ perspective on designing such a dashboard: which obstacles did they encounter? How have they adapted their approach? Do they have any recommendations? These questions would be of interest to the scientific community. If such changes are implemented, I would be happy to update my review. **Response to the Reviewer** *Datasets and dashboards* We have clarified and expanded on the datasets used for developing the dashboards: Supplementary Table 2: Dataset for the Objective 1 dashboard. Supplementary Table 3: Dataset for dashboards developed for Objective 2. Supplementary Table 4: Dataset for creating the Objective 3 dashboard. These datasets have been made publicly available for transparency and reproducibility via GitHub at https://github.com/so13839/Team-8952-Vivli-AMR-Competation/tree/main/data. References 30 to 32 in the revised manuscript now correspond to these datasets. *Impact of the Work* We have revised the “Impact of the Work” section to provide greater emphasis on the dashboards' usability and significance:   The GIS web-based dashboards developed for the ATLAS dataset, covering 83 countries, and the dashboards for Kenya, Uganda, and simulated data from Kampala provide essential tools for exploring and analyzing AMR data. These dashboards are designed to offer a user-friendly, interactive interface tailored to researchers, technicians, and healthcare professionals. The dashboards allow filtering and segmenting of data by key variables, such as country, species, sample sources, and antibiotic resistance profiles. For instance, the Uganda and Kenya dashboards highlight resistance patterns for *E. coli* and visualize data distributions by sample source, year, and resistance levels for antibiotics such as Ampicillin, Levofloxacin, and Imipenem. In Kampala, the simulated dataset provides a focused view, enabling users to identify areas with limited data and prioritize research. Missing data is clearly labeled as “No Information,” helping to highlight gaps requiring further investigation. This interactive approach offers significant improvements over traditional spreadsheet analysis by uncovering spatial and temporal trends efficiently and enabling informed decision-making. *Conclusions and Limitations of the Study* We have rephrased the conclusions to emphasize the dashboards’ potential while addressing their limitations: The interactive GIS dashboards developed using Esri ArcGIS and Shiny represent a significant improvement over spreadsheets, enabling efficient exploration of geographic and temporal trends in AMR data. However, several challenges were encountered. For Esri ArcGIS dashboards, these included the platform's complexity, steep learning curve, and the large dataset size, which required optimization strategies such as data aggregation to improve performance. Limited customization options and the high subscription costs of Esri tools also posed barriers, especially for smaller organizations. Shiny dashboards presented challenges such as the steep learning curve of R programming, deployment complexities, and integration with external APIs. Managing server configurations for platforms like ShinyApps.io required advanced technical skills. Additionally, the study highlights the limitations in AMR data availability, particularly in Africa. Data scarcity, limited sample sizes, and inconsistencies in data collection practices across Uganda and Kenya reduce the dashboards’ reliability. To address these challenges and enhance the dashboards’ effectiveness, we recommend standardized data collection practices, investment in laboratory infrastructure. These steps will ensure the dashboards’ full potential is realized in combating AMR in Africa.

**Reviewer comments**

**Introduction**

 The Introduction can be refocused on the topic of the study – for example, although I agree they are important, the concepts of One Health and antibiotic consumption in animals are not directly relevant in this work. Instead, it would be more relevant for you to introduce GIS dashboards in greater detail – why did the OHE-AMURS work so well? Are there any limitations? How could dashboards be useful in Nepal? By focusing on GIS dashboards before the Objectives section, the scope of the article will be much clearer for readers. **Response to the Reviewer** We have revised the manuscript by removing references to One Health and antibiotic consumption in animals. Instead, we have introduced Geographic Information Systems (GIS) and an Interactive GIS Dashboard as central components of the study. Additionally, we have addressed all the reviewer’s comments in the Introduction, specifically within paragraphs 2 to 5 of the revised manuscript.

**Reviewer comments** **Objectives**

 It is not clear here what you mean by “descriptive statistical analysis”. If this refers to the beginning of your Results section, it is a bit hard to follow, as it explains the conclusions before the results. I understand you may be describing your exact work process during the Vivli Data Challenge, but for readers this will not be clear. You could simply state here that you observed data gaps in African countries and that this motivated your three objectives.

 Throughout: you used the ATLAS dataset in your analysis, so please correct any references to the data accordingly. For example, “the Vivli dataset” is not accurate, since Vivli only facilitate access to the data, but do not own it.

 Objective 3 needs to be clarified. It is not obvious what you mean by “simulated dataset” here, how this may be useful, and what is the link with Kampala, Uganda.  

**Response to the Reviewer** We have removed the descriptive statistical analysis and highlighted the observation of data gaps in African countries in the revised manuscript. Additionally, the Vivli dataset has been replaced with the ATLAS dataset. Objective 3 has been rewritten as: 'To demonstrate the potential usefulness and design of a dashboard that facilitates the analysis of antimicrobial resistance (AMR) patterns in Kampala, Uganda.'"

**Reviewer comments**

**Methods**

 Further information needs to be provided on the ATLAS dataset which you used to inform the dashboards, otherwise readers cannot understand the context and generalisability of the work. For example: how were isolates collected, what is the purpose of this data, which variables does it contain, which biases could it be exposed to... This has been discussed in previous articles which I have referenced below.

**Response to the Reviewer** We have incorporated a discussion of the ATLAS dataset in the revised manuscript, detailing the methods used for isolate collection and referencing previous studies that analyzed this data. Please refer to the section titled Pfizer ATLAS Dataset on page 4 of the revised manuscript.

**Reviewer comments**

**Results**

 Here already, you should adjust the phrasing on the usefulness of GIS dashboards. Here, since you do not directly demonstrate the usefulness of dashboards, you can only discuss their potential value. For example, “is a valuable tool for researchers, policymakers and healthcare professionals” is not supported by the analysis.

**Response to the Reviewer** We have carefully revised the section as per your feedback. The updated section can now be found on page 6 of the revised manuscript. This revision incorporates the necessary clarifications and aligns with the overarching goals of the study. We believe these changes enhance the manuscript's clarity and relevance. If additional adjustments or further clarifications are required, we are happy to address them.  

**Reviewer comments**

 In Objective 2, it is not clear what you mean by “comparative analysis and focused investigations”.  

**Response to the Reviewer** We have carefully addressed your comment and have rewritten and clarified the content in Objective 2, which can now be found on page 6 of the revised manuscript.

**Reviewer comments** For Objective 3 here again it is very unclear what the “simulated dataset” corresponds to and how it was generated. If it is simulated, then interpreting susceptibility results for *K. aerogenes* does not make sense. If the point here is to demonstrate the potential usefulness and design of a dashboard using this type of data, then it should be presented as such.  

**Response to the Reviewer** We have revised the objective to clarify that the simulated dataset is intended to demonstrate how the dashboard could be applied to Kampala, Uganda. The Shiny dashboard created using this simulated data is for illustrative purposes only. The primary intention is to showcase the functionality and potential application of the dashboard. We have included this clarification in the revised manuscript, which can be found on page 7.

**Reviewer comments**

**Discussion**

 A major counter-argument to the use of GIS dashboards is that researchers interested in in-depth analysis would simply obtain the underlying data themselves, rather than use the dashboard. In that sense, you should better discuss the usefulness of dashboards: to whom and how would they be useful exactly? Do you have any examples of dashboards being used in analyses? Are there any previous studies which discussed the potential benefits of GIS dashboards for AMR surveillance? (or for any other surveillance)  

**Response to the Reviewer** In response to your suggestion, we have emphasized the advantages of using dashboards over spreadsheets, particularly in the context of Objectives 1 to 3. Dashboards offer enhanced data visualization, real-time updates, and more intuitive decision-making compared to traditional spreadsheet approaches. For example, Kenya’s One Health AMR Surveillance System (OHAMRS) integrates human and animal health data to visualize AMR patterns across various sectors, making it easier to detect and respond to emerging threats. Similarly, Switzerland’s Swiss Pathogen Surveillance Platform (SPSP) leverages genomics and metadata for effective outbreak monitoring and data sharing. These case studies demonstrate the value of dashboards in surveillance and their ability to improve the efficiency and accessibility of complex data sets. References 15 and 16 in the revised manuscript have been updated to reflect these examples.

**Reviewer comments** As mentioned previously, there are several statements in your Discussion which are not supported by your analysis, such as “The dashboard acts as a centralized hub, aggregating and organizing these datasets for easy access and analysis.” which is not the case here since (if I understood correctly) you only used the ATLAS dataset; “complex queries” are not demonstrated since you present only filtering commands; “examine the impact of different policies or interventions in specific countries, evaluate the effectiveness of existing strategies” policies and interventions are not implemented in the dashboard; “healthcare outcomes” are not included in the dashboard.  

**Response to the Reviewer** We have revised the **Impact of the Work** and **Conclusions sections** to better reflect the findings of our study. The updated content can be found on pages 9 to 10 of the revised manuscript. We believe these revisions provide a clearer and more accurate representation of the implications of our results.

# Reviewer report: Reviewer response for version 1

**DOI:** 10.21956/wellcomeopenres.23245.r88572

**Published:** 2024-08-08

**Authors:** Dov Stekel

**Affiliations:** 1 University of Nottingham, Nottingham, England, UK; 2 University of Johannesburg, Auckland Park, Johannesburg, South Africa

This is a very interesting report and the idea of an interactive GIS-enabled AMR tool is very good and has my support.

 The most important problem however is the lack of availability of the dashboard creation code and the synthetic data from Objective 3. It is very difficult to see how this idea could be of general use without access either to the code or to the interface: indeed I would expect under FAIR principles there to be much greater availability of the methods.

**There are a few minor comments about the way it is written:**

- Methods in Abstract: please rewrite the section to avoid the terms "Objective 1" etc: those terms read like a grant application or project plan and don't really fit the report of a published article.
- Introduction first paragraph: authors refer to both UTIs and poultry systems - which is fine (if a bit abrupt). It would be helpful if the authors were to introduce the One Health framing first, because then the link between the human and agricultural aspects would be more logical.(I note that One Health is referred to in the next paragraph - what is needed is a short description of the idea in paragraph 1, with citation, and perhaps justification as to why it is especially important in sub-Saharan Africa (and lower income countries more generally  - as implied by the reference to Nepal))
- Introduction - penaultimate paragraph reads a bit strange - I would suggest deleting that - it is OK to reference the project abstract - but really what we are looking for is a description of the research, not the details of the Vivli data challenge.
- In Objective 3, the phrase "Specifically, let’s focus" is too colloquial and needs modifying.

Is the work clearly and accurately presented and does it cite the current literature?

Yes

If applicable, is the statistical analysis and its interpretation appropriate?

Yes

Are all the source data underlying the results available to ensure full reproducibility?

No

Is the study design appropriate and is the work technically sound?

Partly

Are the conclusions drawn adequately supported by the results?

Yes

Are sufficient details of methods and analysis provided to allow replication by others?

No

Reviewer Expertise:

Computational Biology, especially in One Health approaches to AMR

I confirm that I have read this submission and believe that I have an appropriate level of expertise to state that I do not consider it to be of an acceptable scientific standard, for reasons outlined above.

# Response

**Published:** 2024-08-25

**Authors:** Stephen Opiyo

**Affiliations:** Patira Data Science, LLC, Westerville, Ohio, USA

**Dear Dr. Stekel, ** 

 Thank you very much for taking the time to review our manuscript. We sincerely appreciate your valuable feedback. We will respond to each of your comments thoroughly. For clarity, your comments will be highlighted in red, while our responses will be indicated in blue.   Kind regards, 

 Stephen **Dov** **Stekel**, University of Johannesburg, Auckland Park, Johannesburg, South Africa; University of Nottingham, Nottingham, England, UK This is a very interesting report and the idea of an interactive GIS-enabled AMR tool is very good and has my support.

**Reviewer:**

 The most important problem however is the lack of availability of the dashboard creation code and the synthetic data from Objective 3. It is very difficult to see how this idea could be of general use without access either to the code or to the interface: indeed I would expect under FAIR principles there to be much greater availability of the methods.

**Response to the Reviewer:**

 We have successfully developed an R Shiny dashboard for Objective 3 and uploaded the corresponding data to GitHub. However, we were unable to develop R Shiny dashboards for Objectives 1 and 2 due to the unavailability of third-party data. Additionally, we cannot provide the code for creating dashboards for Objectives 1 and 2, as they were built in the cloud using Esri’s Graphical User Interface (GUI), which does not generate code for cloud-based dashboards. Nevertheless, the links to these dashboards are available at (https://patira.maps.arcgis.com/apps/dashboards/d89c5b039552464f8f04103f05ac889f ) for Objective 1 and (https://patira.maps.arcgis.com/apps/dashboards/a1324c58e49e4561ac9505936db7743a) for Objective 2, as indicated in lines 407 and 408 of the attached manuscript. *Development of R shiny dashboard for Objective 3*

 We developed R shiny dashboard and uploaded the app into R shiny server at http://167.71.249.72:3838/AMR-Dashboard/.  This app gives same results as was produced by Esri’s dashboard.  Figures 3A and 3B in the attached manuscript explain how the dashboard operates, see lines 249 to 283.  The dashboard provides an interactive visualization of antibiotic resistance data using the R Shiny framework. Users are welcomed with a message guiding them to select a species from a dropdown menu (default: *Staphylococcus aureus*) and download data as a CSV file. The dashboard includes various visualizations such as a Source Distribution plot, a Geographical Distribution map for Kampala, a Samples by Year bar chart (2019-2022), a Gender Distribution pie chart, and Antibiotic Resistance plots for antibiotics like Amikacin and Ampicillin. When " *Klebsiella aerogenes*" is selected, the dashboard updates dynamically to display visualizations specific to this species. Source distribution shows sample locations, with geographical data pinpointing cases in Lugala (Figure 3B). The Samples by Year plot indicates an increase in sample counts over time, and the Antibiotic Resistance charts reveal resistance patterns, such as full resistance to Ampicillin and susceptibility to Amikacin. Data can be exported in CSV format using the "Download Results" button. The “app.R” Shiny application is attached, and you can also find it on GitHub (refer to line 348 of the attached manuscript). The simulated dataset, “Kampala_data.csv,” which is used to create the dashboard, is attached as well. It is also available on GitHub within the `data` folder (see lines 267-286 and line 412 of the attached manuscript). Additionally, instructions for installing and using the app are attached and can be found on GitHub (refer to lines 414 and 416 of the manuscript).

**There are a few minor comments about the way it is written:**

**Reviewer:**

- Methods in Abstract: please rewrite the section to avoid the terms "Objective 1" etc: those terms read like a grant application or project plan and don't really fit the report of a published article.
**Response to the Reviewer**
- We have rewritten the Methods in the abstract as suggested. See lines 30 to 35 in the attached manuscript.

 
**Reviewer:**

- Introduction first paragraph: authors refer to both UTIs and poultry systems - which is fine (if a bit abrupt). It would be helpful if the authors were to introduce the One Health framing first, because then the link between the human and agricultural aspects would be more logical.(I note that One Health is referred to in the next paragraph - what is needed is a short description of the idea in paragraph 1, with citation, and perhaps justification as to why it is especially important in sub-Saharan Africa (and lower income countries more generally  - as implied by the reference to Nepal))

**Response to the Reviewer:**

- We have introduced one-health and referenced it the manuscript.  See lines 80 to 82 in the manuscript, and the reference in line 360.

**Reviewer:**

- Introduction - penaultimate paragraph reads a bit strange - I would suggest deleting that - it is OK to reference the project abstract - but really what we are looking for is a description of the research, not the details of the Vivli data challenge.

**Response to the Reviewer:**

- We have deleted the section and added the information on the abstract to lines 113 and 114 of the attached manuscript.

**Reviewer:**

- In Objective 3, the phrase "Specifically, let’s focus" is too colloquial and needs modifying.

**Response to the Reviewer:**

 We have modified the phrase.  See lines 174 to 176 in the attached manuscript.

# Reviewer report: Reviewer response for version 1

**DOI:** 10.21956/wellcomeopenres.23245.r82552

**Published:** 2024-05-16

**Authors:** Md.Abdus Salam

**Affiliations:** 1 International Islamic University Malaysia,, Kuantan, Malaysia

The article entitled ‘ Empowering Global AMR Research Community: Interactive GIS dashboards for AMR data analysis and informed decision-making’ is an original, innovative, and evidence-based work. To my opinion, it is a very good work that bridges the information gap and strategies to address global AMR issue in general and Uganda in particular. The research objectives have been achieved and reflected in the results.  Sufficient details of methods and analysis are provided to allow replication by others.  Data sources underlying the results are available and methodologies used are reproducible. As a whole there is no issue with language but there are some typos and minor suggestions as appended below.

 Under ‘Plain language summary’

 I suggest ‘visualize and analyze data’, instead of analyze and visualize in the.

 Under ‘Abstract’

 I suggest to add ‘AMR’ before the word data in the 2 ^nd^ line of ‘Methods’.

 Under ‘Introduction’

 A sustained increase in resistance among commonly used antibiotics in surgical wards of a Ugandan hospital from 2014 to 2018 underscores the need for ongoing monitoring, surveillance, and infection prevention and control practices.

 The above sentence should be included under the first paragraph of ‘Introduction’.

 However, accessing this data necessitated the submission of a 300-word abstract. The team submitted an abstract

 I suggest to replace ‘this’ with ‘these’.

 Under ‘Results’ (2 ^nd^ paragraph)

 healthcare professionals to analyze and explore data

 I suggest to change as to explore and analyze for the above sentence.

 The limitation(s) has not been mentioned in the ‘Conclusion’, so I checked ‘partly’ for question #6.

 I would like advice to address the typos and  to accommodate other suggestions for better clarity of the article.

Is the work clearly and accurately presented and does it cite the current literature?

Yes

If applicable, is the statistical analysis and its interpretation appropriate?

Yes

Are all the source data underlying the results available to ensure full reproducibility?

Yes

Is the study design appropriate and is the work technically sound?

Yes

Are the conclusions drawn adequately supported by the results?

Partly

Are sufficient details of methods and analysis provided to allow replication by others?

Yes

Reviewer Expertise:

NA

I confirm that I have read this submission and believe that I have an appropriate level of expertise to confirm that it is of an acceptable scientific standard, however I have significant reservations, as outlined above.

# Response

**Published:** 2024-08-25

**Authors:** Stephen Opiyo

**Affiliations:** Patira Data Science, LLC, Westerville, Ohio, USA

Dear Dr. Abdus Salam,

 Thank you very much for taking the time to review our manuscript. We sincerely appreciate your valuable feedback. We will respond to each of your comments thoroughly. For clarity, your comments will be highlighted in red, while our responses will be indicated in blue. Kind regards, 

 Stephen **Response to** **Md.Abdus Salam, International Islamic University Malaysia,, Kuantan, Malaysia** The article entitled ‘ Empowering Global AMR Research Community: Interactive GIS dashboards for AMR data analysis and informed decision-making’ is an original, innovative, and evidence-based work. To my opinion, it is a very good work that bridges the information gap and strategies to address global AMR issue in general and Uganda in particular. The research objectives have been achieved and reflected in the results.  Sufficient details of methods and analysis are provided to allow replication by others.  Data sources underlying the results are available and methodologies used are reproducible. As a whole there is no issue with language but there are some typos and minor suggestions as appended below.

**Reviewer:**

 Under ‘Plain language summary’

 I suggest ‘visualize and analyze data’, instead of analyze and visualize in the.

**Response to the Reviewer:**

 We have change to ‘visualize and analyze data’. See line 54 of the attached manuscript. **Reviewer:**

 Under ‘Abstract’

 I suggest to add ‘AMR’ before the word data in the 2 ^nd^ line of ‘Methods’.

**Response to the Reviewer:**

 We have added ‘AMR’ before the word data.  See line 30 of the attached manuscript. **Reviewer:**

 Under ‘Introduction’

 A sustained increase in resistance among commonly used antibiotics in surgical wards of a Ugandan hospital from 2014 to 2018 underscores the need for ongoing monitoring, surveillance, and infection prevention and control practices.

 The above sentence should be included under the first paragraph of ‘Introduction’.

**Response to the Reviewer:**

 We have moved “A sustained increase in resistance among commonly used antibiotics in surgical wards of a Ugandan hospital from 2014 to 2018 underscores the need for ongoing monitoring, surveillance, and infection prevention and control practices.” to the first paragraph.  See lines 81 to 83 in the attached manuscript.

**Reviewer:**

 However, accessing this data necessitated the submission of a 300-word abstract. The team submitted an abstract

 I suggest to replace ‘this’ with ‘these’.

**Response to the Reviewer:**

 We have replaced ‘this’ with ‘these’. See line 100 of the attached manuscript.

**Reviewer:**

 Under ‘Results’ (2 ^nd^ paragraph)

 healthcare professionals to analyze and explore data

 I suggest to change as to explore and analyze for the above sentence.

**Response to the Reviewer:**

 We have change to ‘explore and analyze ‘. See line 180 of the attached manuscript.

**Reviewer**

 The limitation(s) has not been mentioned in the ‘Conclusion’, so I checked ‘partly’ for question #6.

**Response to the Reviewer:**

 We have added the paragraph below about the limitations of the study to the Conclusion. See lines 236 to 242 of the attached manuscript.

**Limitations:**

 “However, the research confronts notable challenges, particularly the scarcity of comprehensive AMR data in Africa. Limited sample sizes, such as those in Uganda and Kenya, may not accurately reflect the full spectrum of resistance patterns. Furthermore, inconsistent data collection and reporting across Africa reduce the reliability and generalizability of dashboards, particularly for Uganda and Kenya. The study underscores the necessity for standardized data collection, enhanced laboratory capabilities, and increased investment in healthcare infrastructure to fully leverage dashboards in the fight against AMR in Africa”.
